# Up-regulation of calreticulin in mouse liver tissues after long-term irradiation with low-dose-rate gamma rays

**DOI:** 10.1371/journal.pone.0182671

**Published:** 2017-09-20

**Authors:** Lan Yi, Nan Hu, Jie Yin, Jing Sun, Hongxiang Mu, Keren Dai, Dexin Ding

**Affiliations:** 1 Key Discipline Laboratory for National Defense for Biotechnology in Uranium Mining and Hydrometallurgy, University of South China, Hengyang, Hunan, P.R. China; 2 College of Pharmacy and Biological Sciences, University of South China, Hengyang, Hunan, P.R. China; Northwestern University Feinberg School of Medicine, UNITED STATES

## Abstract

The biological effects of low-dose or low-dose-rate ionizing radiation on normal tissues has attracted attention. Based on previous research, we observed the morphology of liver tissues of C57BL/6J mice that received <50, 50–500, and 500–1000 μGy/h of ^137^Cs radiation for 180 d. We found that the pathological changes in liver tissues were more obvious as the irradiation dose rates increased. Additionally, differential protein expression in liver tissues was analyzed using a proteomics approach. Compared with the matched group in the 2D gel analysis of the irradiated groups, 69 proteins had ≥ 1.5-fold changes in expression. Twenty-three proteins were selected based on ≥2.5-fold change in expression, and 22 of them were meaningful for bioinformatics and protein fingerprinting analysis. These molecules were relevant to cytoskeleton processes, cell metabolism, biological defense, mitochondrial damage, detoxification and tumorigenesis. The results from real-time PCR and western blot (WB) analyses showed that calreticulin (CRT) was up-regulated in the irradiated groups, which indicates that CRT may be relevant to stress reactions when mouse livers are exposed to low-dose irradiation and that low-dose-rate ionizing radiation may pose a cancer risk. The CRT protein can be a potential candidate for low-dose or low-dose-rate ionizing radiation early-warning biomarkers. However, the underlying mechanism requires further investigation.

## Introduction

It is widely accepted that high doses of radiation are extremely pernicious to tissues and cells. Epidemiological studies data from A-bomb survivors and other populations exposed to ionizing radiation have verified these effects [1, 2]. These data are largely composed of high-dose and high-dose-rate exposures with mixed radiation types and intrinsic uncertainties in dosimetry [3]. Recently, there has been increased interest in the biological effects of low-dose or low-dose-rate ionizing radiation on normal tissues [4, 5]. Research has shown that the effects of low-dose radiation on cells and tissues are different from those of high-dose radiation [6]. These effects probably include cell proliferation, chromosomal aberrations, cell-cycle disturbance, DNA damage, cell death and point mutations [7, 8]. Currently, the radiation protection of risk assessment assumes that compared with high-dose exposures, the same level per unit absorbed dose or effective dose is caused by low-dose and low-dose-rate exposures [9]. However, the molecular events related to specific radiation doses have not been determined. Thus, it is important to develop a low-dose-rate biomarker for radiation protection.

Microarray and proteomics are two effective methods for studying biomarkers. Microarray expression analysis provided an integrated view of the biological effects of low-dose ionizing radiation [10]. In vivo experiments on mouse kidney or liver tissues resulted in obvious differences between low- and high-dose irradiation [11, 12]. These studies indicated several common genes that are affected by low-dose exposures at different points in time. Identification of the proteins responsible for the adaptive reactions to radiation would increase our understanding of the molecule mechanisms relevant to protection against low-dose-rate and low-dose radiation. Quantitative proteomics techniques have been well used to determine tumor markers [13, 14]. Through proteomics analysis, our previous research identified differentially expressed proteins in liver tissues of mice that had received ^137^Cs radiation for 90 d [15]. The purpose of this study was to determine biomarkers of radiation exposure using quantitative proteomics for use as early warning detection of radiation pollution. Hence, we used mass spectrometry analysis, bioinformatics tools, and two-dimensional electrophoresis (2-DE) to explore how low-dose-rate radiation from ^137^Cs over 180 days affected protein expression in the mouse liver when compared with that of control mice. Our research may tremendously affect health risk assessment of low-dose-rate radiation exposure, which is a crucial component of radiation protection.

## Materials, design, and methods

### Reagents and materials

The 2-D Quant Kit and the solid-phase dry pH gradient gel strips were obtained from Amersham Pharmacia (Tokyo, Japan) and from Bio-Rad (California, USA). We also used the PDquest two-dimensional gel spectrum analysis software. The peptide calibration standard II was the product of Bruker Daltonics Inc. (Massachusetts, USA). Bioinformatics software came from Applied Biosystems (Foster, USA), and mass spectrometry reagents were from Sigma. The Mascot peptide mass fingerprinting (PMF) database query software and Mascot Distiller mass spectrum signal peak recognition software were obtained from Matrixscience. The catalase (CAT), glutathione S-transferase P1 (GSTP1), and Calreticulin (CRT) antibodies were from Santa Cruz Biotechnology (California, USA). The total RNA extraction kit was from Omega Company (Norcross, USA), and the RT kit was obtained from Promega; all other generic reagents came from Shanghai Biotechnology.

### Mice and irradiation

We use C57BL/6J male mice that were obtained from the Hunan Slack King Laboratory Animal Co. Ltd. (Changsha, China). They were irradiated and bred in the Animal Department of the University of South China. At 6 weeks of age, 40 mice were randomly divided into four groups. Three groups of mice were exposed to whole-body radiation with a 137Cs gamma radiation source at dose rates of <50, 50–500, and 500–1000 μGy/h for 180 days (22 h exposure per day). A gamma ray detector was used to detect dose rates (Fuzhou, China). A group of nonirradiated control mice was used for each experiment. The unirradiated matched group was also bred in the different cages of the same room with the irradiated groups, a lead brick was used as a Barrier. The dose rate of the unirradiated matched group is about <0.2μGy/h. The mice were group housed throughout the experiments, and the animals were sacrificed via cervical dislocation after 180 days of exposure. The livers were harvested. We divided the edge of liver tissues into three parts: one used for 2D gel electrophoresis, another was used for histology, and the third was for RT-PCR and westerns. Those parts for histology were fixed and embedded in paraffin. Others were washed with PBS, and snap frozen at -80℃ for later use. All experimental procedures were conducted in conformity with institutional guidelines for the care and use of laboratory animals, and the protocols were approved by the Institutional Animal Care and Use Committee in University of South China, Hunan, China.

### Protein extraction and quantification

A mortar and pestle were cooled by liquid nitrogen before the frozen livers were homogenized to powder. The tissues of different mice of the same group were mixed together before extracting proteins. The tissue powders were resuspended in rehydration lysis buffer, and the mixture was placed on a shaking table at 4°C for 1 h before lysis by sonication, centrifugation, and supernatant collection. Supernatants extracted from the same group were mixed together. The proteins were quantified using the 2D Quant Kit, and the supernatants were stored at −20°C.

### 2-DE, staining, and image analysis

One thousand micrograms of total protein was extracted from the mouse livers from the irradiation and matched groups. Solid-phase isoelectric focusing (IEF) with a pH gradient followed by second-phase SDS-PAGE electrophoresis was then used. After electrophoresis, the gels were silver-stained by Coomassie brilliant, and PD Quest software was used to scan for background subtraction. The resulting images were analyzed by software for spot detection, comparisons, quantification and statistical analyses. Each spot was analyzed with six gel images: three for control group and three for irradiation groups at different doses.

### In situ protein digestion and MALDI-TOF mass spectrometry

Using a Powerlook1100 to achieve gel images at 300 dpi (dots/in), the results were then analyzed using the GE Healthcare (Stockholm, Sweden) ImageMaster™ 2D Platinum 5.0 software and UMAX (Taipei, China). The spots were detected using the 2D Elite Detection Program, and the spot volume was calculated relative to the background values used for normalization. By comparing the spot volume to the total volume in the 2D gel, the volume percent of each spot was determined. Using Student’s t-test to identify the differentially expressed protein spots between the control and experimental groups, a fold-change of ≥ 1.5 and values of p<0.05 were considered statistically significant. Before mass spectrometry analysis, we subjected the protein spots to enzymatic hydrolysis followed by destaining and drying. Two standards were used to obtain the final PMF and calibrate the spectrum. The external standard used was the Peptide calibration standard II. The internal standard was the matrix peak and trypsin auto-degradation ionic peak.

### Bioinformatics analysis

GPS Explorer software version 3.6 (Applied Biosystems) was used to conduct peak list generation and database searching against the International Protein Index (IPI) mouse protein database with the Mascot database search algorithm (version 2.1; Matrix Science Ltd). Protein identification by PMF using Mascot was carried out using parameters similar to those used for the IPI mouse database. The IPI mice protein and Mascot database search engine were used to determine gene ontology categories, gene names and protein functions. Proteins with scores > 55 and located outside of the green shadow were considered to be meaningful proteins.

### Hematoxylin and eosin (H&E) staining of liver tissues

Rehydrated tissue sections were fixed in either alcohol or an aldehyde-based fixative. The slides were immersed in H2O with agitation for 30 sec and then dipped in Mayer’s hematoxylin for 30 sec with agitation. The slides were rinsed for 1 min in H2O and then stained with 1% eosin Y solution with agitation for 10–30 sec. The sections were dehydrated with two kinds of alcohol for 30 sec each. Xylene was used to extract the alcohol. One or two drops of mounting medium were added, and the sections were covered with a cover slip.

### Real-time PCR verification

In our previous reports, GSTP1 and CAT proteins were up-regulated in all three experimental groups after 90 days of exposure [15]. Considerable evidence in recent years has suggested that CRT is involved in tumorigenesis. Thus, real-time PCR and western blot analysis were used to select CRT proteins for validation.

Glutathione S-transferase P1 (GSTP1), catalase (CAT), and calreticulin (CRT) proteins were selected for verification by western blot analyses and real-time PCR. Sybrgreen quantitative real-time PCR was used to measure GSTP1, CAT and CRT gene expression in liver tissues of mice receiving different irradiation dose rates. GAPDH was used as an internal control. All mice in a group were divided over 3 pools (10 mice in a group, divided into 4+3+3). The tissues of different mice of the same pools were mixed together before extracting RNA.

Premier 5.0 software was used to design all primer sequences, which were synthesized by the Takara Biotechnology Company (Shiga, Japan). The primer sequences are shown in Table 1:

**Table 1:** The primer sequences for CRT, CAT, GSTP1 and GAPDH.

Gene	Primer sequence	length
GAPDH	F: 5' TGGCAAAGTGGAGATTGTTGCC 3' R: 5' AAGATGGTGATGGGCTTCCCG 3'	156 bp
CRT	F: 5′GGAAGATGAGGAGGAAGATGTC-3′ R: 5′CAGGAAGGAGAGCAGATGAAAT-3′	212 bp
GSTP1	F: TCTACGCAGCACTGAATCCG R: GCCCTCGAACTGGGAAGTAG	72 bp
CAT	F: GCGGATTCCTGAGAGAGTGG R: TGTGGAGAATCGAACGGCAA	145 bp

According to the manufacturer’s directions, we used a Total RNA kit II to extract total RNA, and the NanoDrop® ND-1000 to measure RNA purity and concentration. Reverse transcription was then used to obtain the cDNA. Two micrograms of total RNA was heated with 1 μL of 0.5 μg/μL oligo (dT)18 to 70°C for 3 min. After cooling to 37°C, the mixture was incubated for 10 min. This reaction required 4μL of 2.5 mM dNTP mixture and 2μL of 10x RT buffer, 200 units of Moloney murine leukemia virus reverse transcriptase, and 1 μL of RNase inhibitor. First, the mixture was incubated for 1 h at 37°C and then for 5 min at 95°C and finally cooled on ice. For real-time PCR, the reactions were conducted in a 25 μL mixture including 2.5 μL of 2.5 mM dNTP, 1.5 μL of MgCl_2_, 2.5 μL of 10 × PCR buffer, 1 μL of Primer1, 1 μL of Primer2, 1 μL of cDNA, 2× 1 unit TaqMasterMix, and 10,000-fold diluted Sybrgreen. The PCRs were first heated for 5 min at 95°C and then 10 s at 95°C, 15 s at 59°C, and 20 s at 72°C for 40 cycles. Heating samples from 72°C to 99°C provided a melt curve analysis.

### WB verification

Total proteins from match group and irradiated mice were quantified by BCA protein quantification assay. Total protein (50 μg) was mixed with loading buffer at a 5:1 ratio. We denatured proteins by boiling samples for 5 min at 100°C and then separated the proteins on 10% sodium dodecyl sulfate-polyacrylamide gel electrophoresis and transferred them onto a polyvinylidene difluoride (PVDF) membrane blocked with 0.1% Tween20 (TBS-T) containing 5% nonfat dry milk for 2 h at room temperature. After blocking, the first antibody was incubated for 2 h at 37°C. We used TBST to wash the PVDF five times (10 min per wash) and then added the secondary antibody (1 h), and enhanced chemiluminescence (ECL) was used to detect antibody binding. Western blots results were quantified by chemical luminescence reagent.

### Statistics

The results were presented as the mean±standard deviation (SD). All statistical analysis was performed with the SPSS statistical software, version 18.0. The differences between groups were analyzed using the t-test, and the statistical significance level was defined as two-sided P <0.05.

## Results

### Morphological changes in liver tissues of mice that received different irradiation dose rates

**Fig 1:**
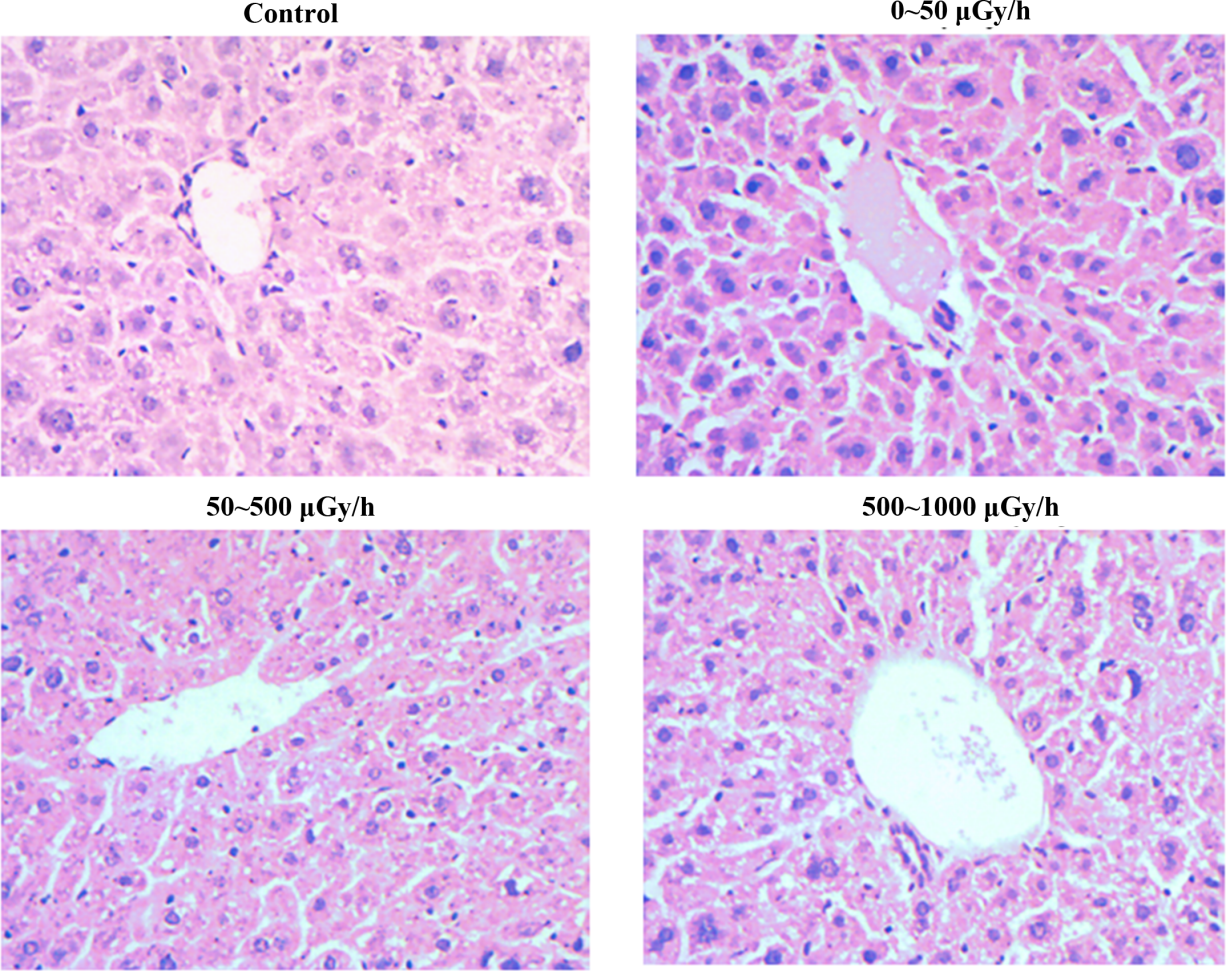
Morphology of liver tissues of mice received different irradiation dose rates (HE×400).

As shown in Fig 1, compared with the matched group, the central vein was slightly dilated in the < 50 μGy/h irradiated group, and other pathological changes to the liver tissues were not observed. In the 50–500 μGy/h and 500–1000 μGy/h groups, central vein dilation was obvious, the necrosis and inflammation of liver tissues were prominent, and there were also some steatosis and interstitial fibrosis.

### Comparative proteomics analysis of unirradiated and irradiated mice by 2-DE

The components of total proteins of mouse were separated by 2-DE. The IPG strip was 24 cm at PH 3–10. Coomassie brilliant blue G-250 was used for staining, and the image results were obtained with a UMAX scanner. All of the results were repeated at least 3 times, and consistency was confirmed. In each of the gels, we found at least 600 spots. Compared with the matched group, there were 20, 21, and 28 spots with more than 1.5-fold changes compared with the 500–1000 μGy/h, 50–500, and <50 groups, respectively.

### Mass spectrometry and bioinformatics analyses of differential protein spots

**Fig 2:**
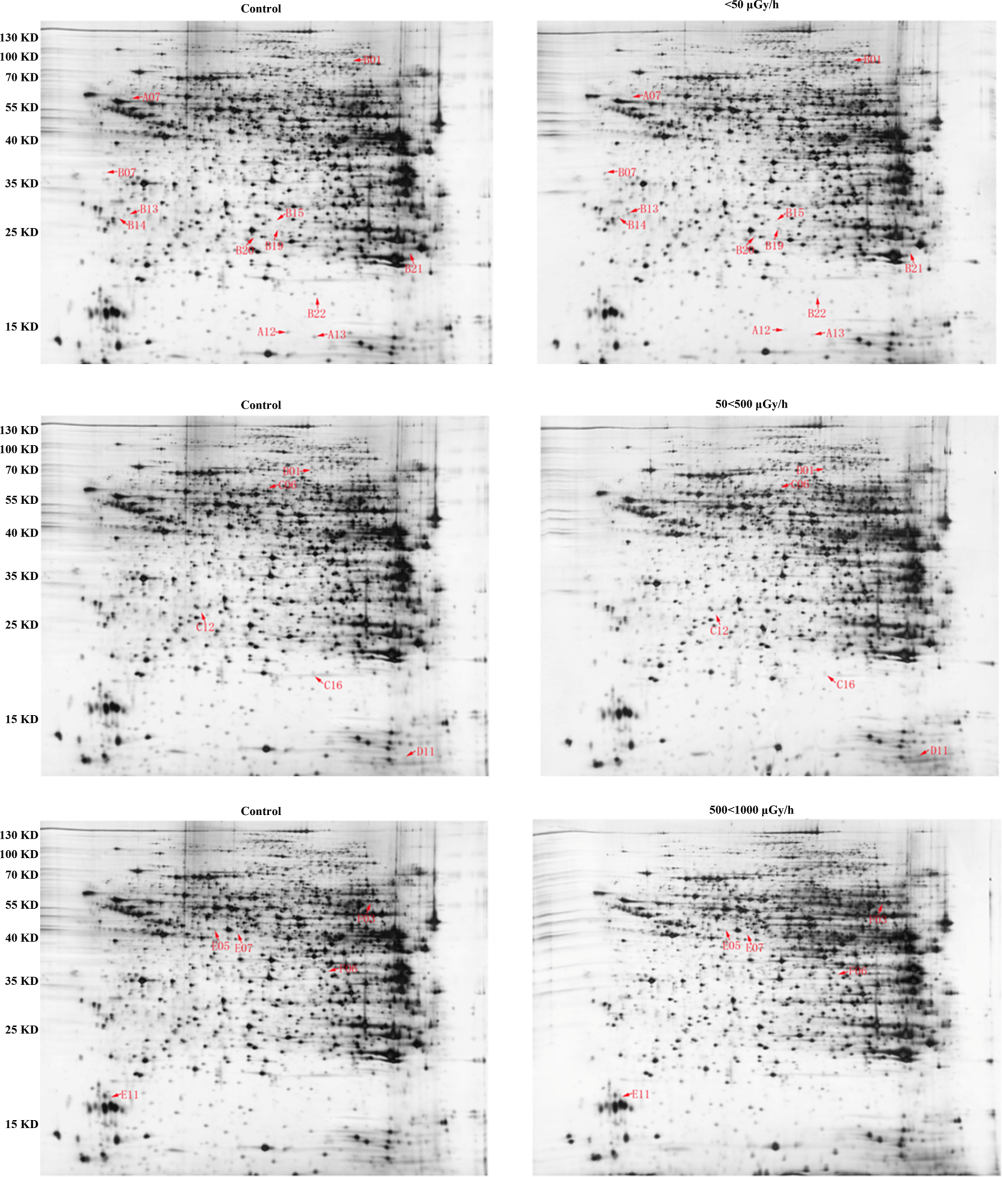
2-DE with Coomassie Blue staining of total protein in the matched group and three irradiated groups.

**Fig 3:**
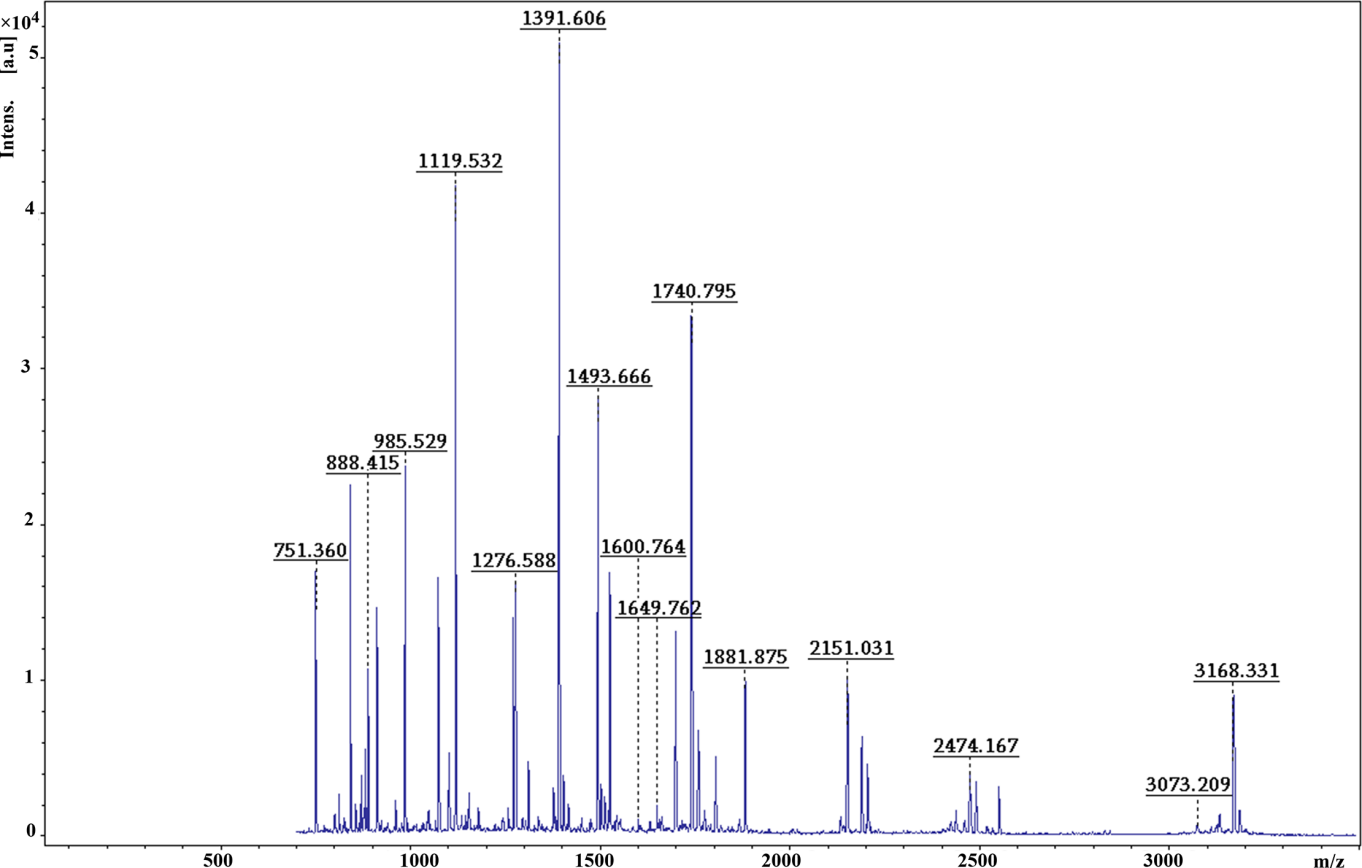
PMF of protein spot B07 obtained from MALDI-TOF- MS.

Twenty-three spots (≥2.5-fold changes in expression with obvious error dots removed) were selected and analyzed with MALDI-TOF-MS. Database searching was used to identify the proteins. Fig 2 shows twenty-two of these spots, and PDQuest software provided accurate spectral information. Fig 3 shows that we used MALDI-TOF-MS to obtain the image results of spot no. B07. The SWISS-PROT protein databases and NCBI were used to analyze 22 protein spots, and Protein spot no. B07 was identified as CRT with a score of 200 and a sequence coverage of 37%. The scores of 22 protein spots were more than 55, and the results are displayed in Table 2.

**Table 2:** Differentially expressed proteins identified by MALDI-TOF-MS.

Spot	Entry name	Protein identified	Mr (Da)	IP	Score	Sequence coverage	Function
A07	NUCB1_MOUSE	Nucleobindin-1^#^	53376	4.99	440	57%	Calcium binding protein
A12	COF1_ MOUSE	Cofilin-1^#^	18776	8.22	202	48%	tumorigenesis
A13	DEST_ MOUSE	Destrin^#^	18852	8.14	194	53%	cytoskeleton
B01	CPSM_ MOUSE	Carbamoyl-phosphate synthase [ammonia], mitochondrial*	165711	6.48	118	7%	mitochondrial damage
B07	CALR_ MOUSE	Calreticulin*	48136	4.33	200	37%	Calcium binding protein
B13	ACTG_ MOUSE	Actin, cytoplasmic 2*	42108	5.31	114	36%	cytoskeleton
B14	1433G_MOUSE	14-3-3 protein gamma*	28456	4.8	119	34%	gene transcription
B15	NIT2_ MOUSE	Omega-amidase NIT2*	30825	6.44	173	28%	tumor suppressor
B19	BHMT1_MOUSE	Betaine-homocysteine S-methyltransferase 1*	45448	8.01	155	40%	metabolism
B20	FTHFD_MOUSE	10-formyltetrahydrofolate dehydrogenase*	99502	5.64	282	16%	Catalysis
B21	MMAB_MOUSE	Cob(I)yrinic acid a,c-diamide adenosyltransferase, mitochondrial*	26542	9.32	189	37%	Catalysis
B22	GSTP1_MOUSE	Glutathione S-transferase P 1*	23765	7.68	69	21%	Liver detoxification
C06	HACL1_MOUSE	2-hydroxyacyl-CoA lyase 1^#^	64588	5.89	164	21%	Catabolism
C12	PSME2_MOUSE	Proteasome activator complex subunit 2^#^	27268	5.54	76	24%	Immunoproteasome assembly
C16	GPX1_ MOUSE	Glutathione peroxidase 1^#^	22544	6.74	106	36%	Metabolism
D01	CPSM_ MOUSE	Carbamoyl-phosphate synthase [ammonia], mitochondrial*	165711	6.48	74	8%	Mitochondrial damage
D11	H2B1B_MOUSE	Histone H2B type 1-B*	13944	10.31	78	58%	Cytoskeleton
E05	ALBU_ MOUSE	Serum albumin^#^	70700	5.75	99	18%	Stabilizing extracellular fluid
E07	NAGA_ MOUSE	Putative N-acetylglucosamine-6-phosphate deacetylase^#^	43929	5.78	82	13%	Catalysis
E11	MUP2_ MOUSE	Major urinary protein 2^#^	20935	5.04	266	81%	Bind and release pheromones
F03	CATA_ MOUSE	Catalase*	60013	7.72	546	58%	Biological defense
F06	DHE3_ MOUSE	Glutamate dehydrogenase 1, mitochondrial*	61640	8.05	105	26%	Nitrogen and glutamate metabolism

IP: isoelectric point

*Up-regulated compared with the matched group

# Down-regulated compared with the matched group.

### Real-time PCR verification

In this experiment, CRT and GSTP1 were up-regulated in the <50 μGy/h irradiated group, and CAT was up-regulated in the 500–1000 μGy/h irradiated group when compared with the match group.

**Fig 4:**
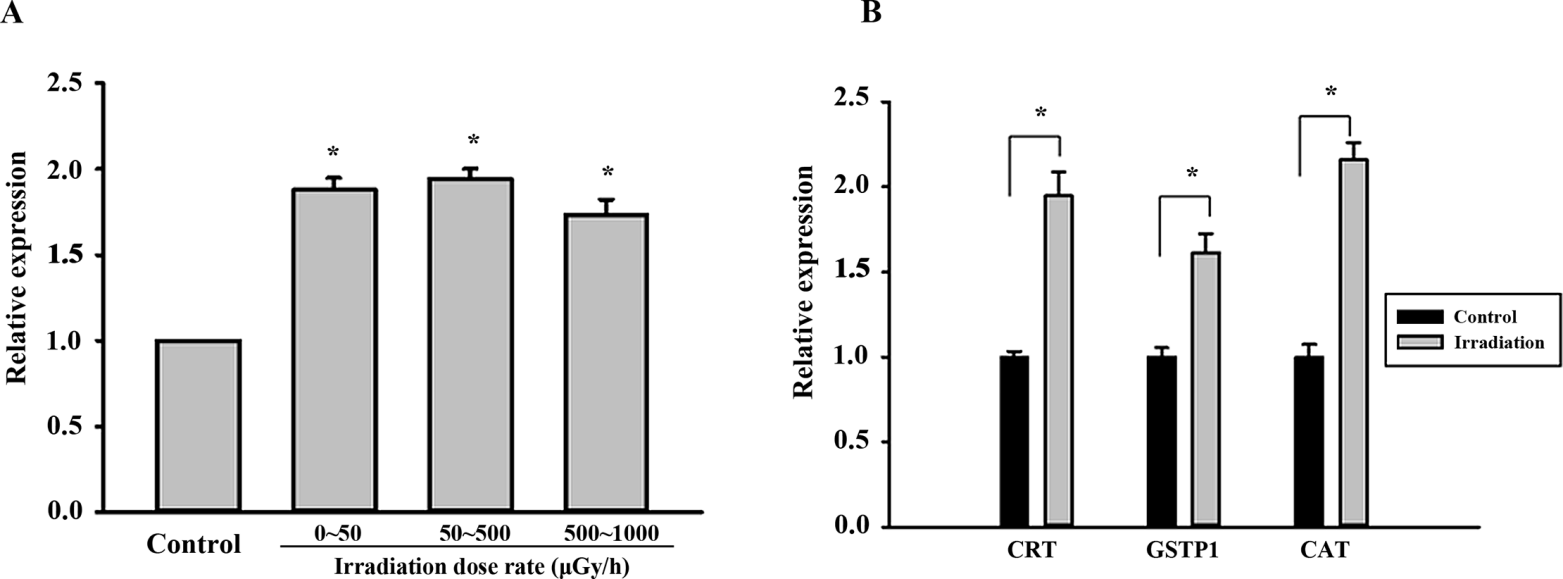
QPCR verification CRT, GSTP1 and CAT gene expression in liver tissues of mice receiving different irradiation dose rates. (A) CRT mRNA expression levels in three different dose rate treated groups. *P < 0.05 vs. control cells. (B) CRT (<50 μGy/h), GSTP1 (<50 μGy/h), CAT (500–1000 μGy/h) gene expression. Values are presented as mean ± SD from three experiments. *P < 0.05 vs. control cells.

As shown in Fig 4A, consistent with the proteomics analysis, the expression levels of CRT mRNA were increased at three different dose rates in the treated groups (P<0.05). GSTP1 (<50 μGy/h) and CAT (500–1000 μGy/h) mRNA expression levels were increased in the experimental group vs. the matched groups (P<0.05) (Fig 4B), which was consistent with proteomics analysis results.

### WB verification

**Fig 5:**
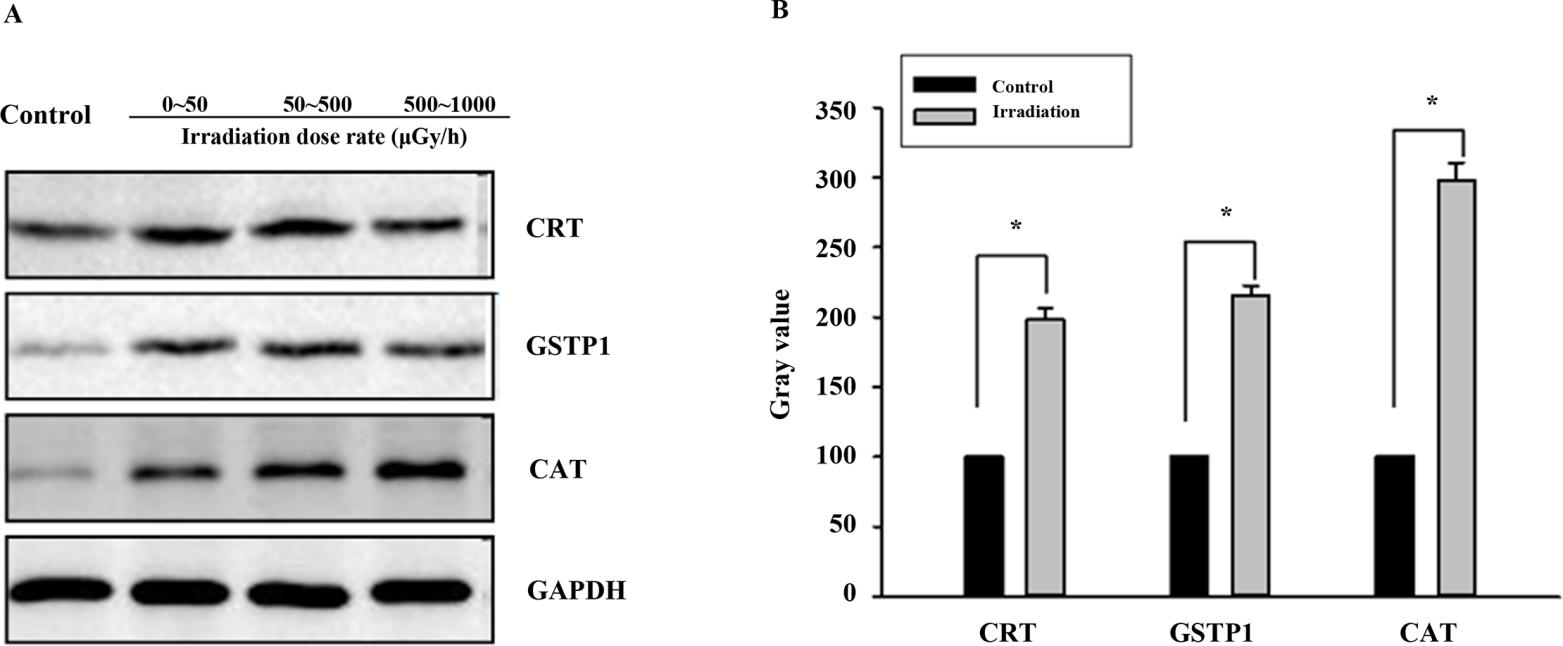
WB verification of CRT, GSTP1 and CAT protein expression in liver tissues of mice receiving different irradiation dose rates. (A) Representative WB analysis of CRT, GSTP1 and CAT expression using GAPDH as the internal control. (B) Quantification of CRT (<50 μGy/h), GSTP1(<50 μGy/h), CAT (500–1000 μGy/h) expression by western blot analysis. Values are presented as mean ± SD from three experiments. *P < 0.05 vs. control cells.

WB analysis was used to verify the CRT, GSTP1 and CAT protein expression levels. After 180 days of irradiation, CRT, CAT and GSTP1 protein expression was elevated compared with that of the matched group (P<0.05) (Fig 5), which was consistent with the analysis results.

## Discussion

The methodology described above has important significance to our research. It has many kinds of effects on normal organisms that received radiation [16]. Analysis of differentially expressed proteins in cells and tissues under different conditions is one of the most effective strategies in determining relevant molecules in the response to low-dose radiation [17]. These differentially expressed proteins can be used as mechanisms of early warning to protect against radiation exposure [18].

According to our previous studies, we observed the morphology of liver tissues of mice that received diverse radiation dose rates for 180 d. Compared with the matched group, the irradiation dose rates and the total dose increased, and the pathological changes in liver tissues were more obvious. These results demonstrated that higher irradiation dose/ dose rates do greater harm to the liver tissues. We also analyzed and compared differentially expressed proteins in the liver tissues of mice that received low-dose-rate ^137^Cs irradiation for 180 d. We selected twenty-three protein spot image results by MALDI-TOF MS analysis. Twenty-two of these were determined successfully. One of the spots may have been too low in intensity to generate high-quality mass spectrometric data and was not successfully identified. In mouse liver tissues, 13 proteins of these 22 proteins relevant to low-dose-rate irradiation were up-regulated, including Calreticulin (CRT), 14-3-3 protein gamma, Omega-amidase NIT2, betaine-homocysteine S-methyltransferase 1,10-formyltetrahydrofolate dehydrogenase, glutathione S-transferase P 1, catalase, and histone H2B type 1-B, and 9 proteins were down-regulated, including nucleobindin-1, cofilin-1, destrin, 2-hydroxyacyl-CoA lyase 1, proteasome activator complex subunit 2, glutathione peroxidase 1, and major urinary protein. These 22 proteins are related to cytoskeleton processes, cell metabolism, biological defense, mitochondrial damage, detoxification and tumorigenesis.

CAT is prevailingly located in the erythrocytes and liver as a crucial enzyme for the biological defense system [19]. GSTP1 is, with many functions, an acrolein-metabolizing enzyme [20]. In our previous reports, GSTP1 and CAT proteins were up-regulated in three experimental groups for 90 days [15]. In this study, CAT and GSTP1 in the irradiation groups also had a higher expression than those in the matched group. These results indicated that the expression level of GSTP1 and CAT in liver tissues had a relationship with the ionizing radiation, and irradiation may induce damage to the tissues or organisms.

It is well known that following ionizing radiation exposure, many researchers have provided fundamental data on the risk to cancer in humans [21]. As is known to all, ionizing radiation may change the expression of proteins and cause pathological changes, such as tumorgenesis [22]. Proteomics analysis has been useful in examining the expression of cancer associated candidate proteins [23]. In this research, we found that CRT expression was enhanced in the liver tissues of mice when the mice were exposed to low-dose-rate radiation. CRT is a multi-process endoplasmic reticulum protein involved in cell adhesion, cell–cell interaction, migration, phagocytosis, integrin-dependent Ca2+ signaling, and immunity [24]. CRT also plays an important role in cell apoptosis, differentiation, and proliferation [25]. Very recently, numerous studies have shown that CRT is associated with several cancers [26], and emerging evidence supports its role in tumor formation and progression depending on the cell type and clinical disease stage [27]. CRT expression was elevated in bladder cancer tissues compared with normal tissues, and alteration of its expression might affect bladder cancer progression in vitro and in vivo [28, 29]. CRT concentrations in the sera of lung cancer patients were higher than those in healthy individuals, and the effect of CRT on the patient cell membranes was related to tumor pathological grade and classification [30]. CRT expression was significantly increased in pancreatic cancer compared with paired non-cancerous pancreatic tissues, and high expression was positively associated with the tumor UICC stage and lymph node metastasis [31]. CRT was also highly expressed in oral squamous cell carcinoma tumor tissues and oral squamous cell carcinoma (OSCC) cell lines [32]. CRT expression was enhanced in the breast cancer cell lines MCF-7 and MDA-MB-231 compared with the normal breast epithelial cell line MCF-10A [33]. Diallyl disulfide treatment tremendously transformed the morphology of human promyelocytic leukemia HL-60 cells and created a vastly time-dependent downregulation of CRT [34]. Recent findings suggest that CRT could be a target for the development of novel therapeutics [35].

It is well known that ionizing radiation at high doses is harmful to the exposed organism. However, biological effects of low-dose or low-dose-rate ionizing radiation remain unclear [36]. Low-dose-rate/low-dose radiation is an injury-related course, which is relevant to numerous elements and procedures, whereas the response of the organism is also relevant to multifarious proteins and numerous signaling pathways [37]. This study reports that CRT is involved in the 180-d radiation-induced stress response in liver tissues of mice. CRT is related to many diseases such as cancer [38]. Our research demonstrates that low-dose or low-dose-rate ionizing radiation may pose a cancer risk. The CRT protein can be a potential candidate for low-dose or low-dose-rate ionizing radiation early-warning biomarkers. However, further research is required to determine the dose-rate dependence of CRT expression and to identify whether it is altered in other parts of the body such as the blood.

This project was sponsored by the Key Project of National Defense Basic Research (No. B3720132001), National Natural Science Foundation of China (No. U1401231), China Postdoctoral Science Foundation (No. 2014M562115), the National Natural Scientific Foundation of China (81400117), the Hunan Provincial Natural Science Foundation of China (2015JJ4042), Hunan Provincial Postgraduate Students Scientific Research Innovation Project (CX2016B473) and the Research Initiation Funding of University of South China for the Returned Scholars from Abroad (No. 2014XQD46).

## Supporting information

S1 File Spot on gel. (DOCX)

S2 File PMF of protein spot from MALDI-TOF- MS. (DOCX)

S3 File Real time PCR. (DOCX)

S4 File Gray value of CRT, GSTP1, CAT. (DOCX)

S5 File Arrive guidelines checklist. (PDF)
