# Supplementary figures and images for: Up-regulation of calreticulin in mouse liver tissues after long-term irradiation with low-dose-rate gamma rays
Source: PLoS One. 2017 Sep 20;12(9):e0182671. doi: 10.1371/journal.pone.0182671 (PMC5607120; doi:10.1371/journal.pone.0182671)

**SPOT ON GEL**

**
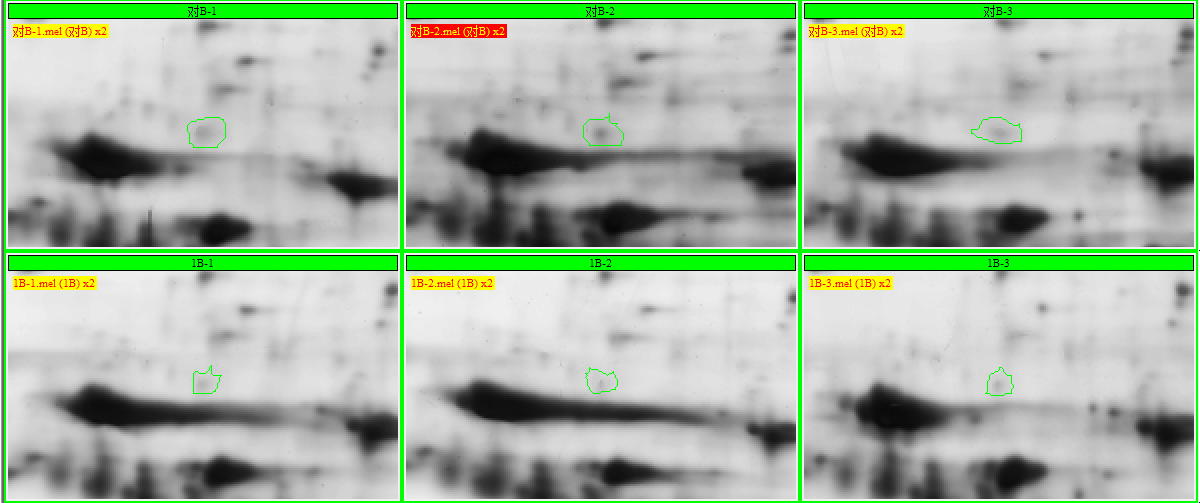
**

**A07**

**
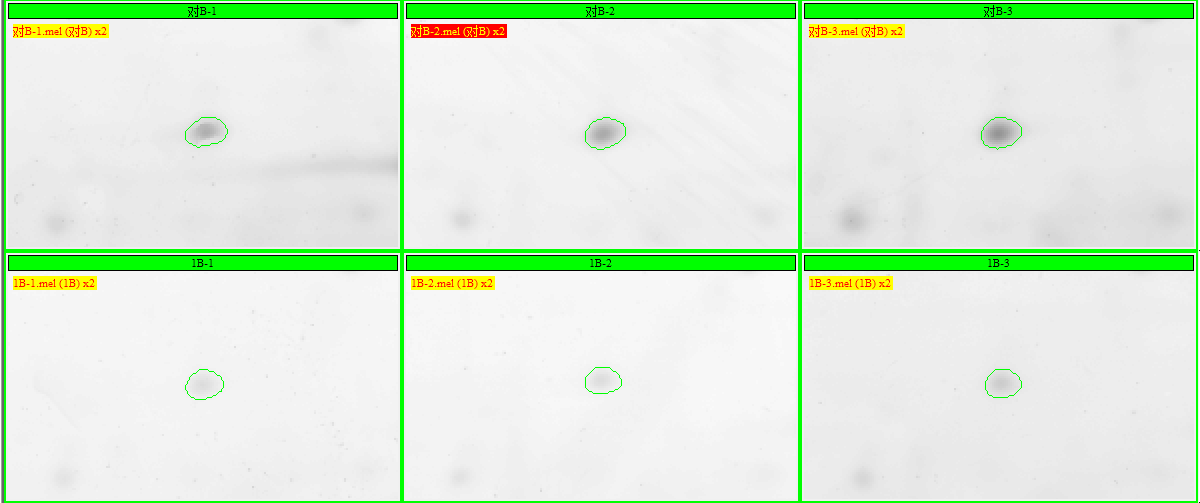
**

**A12**

**
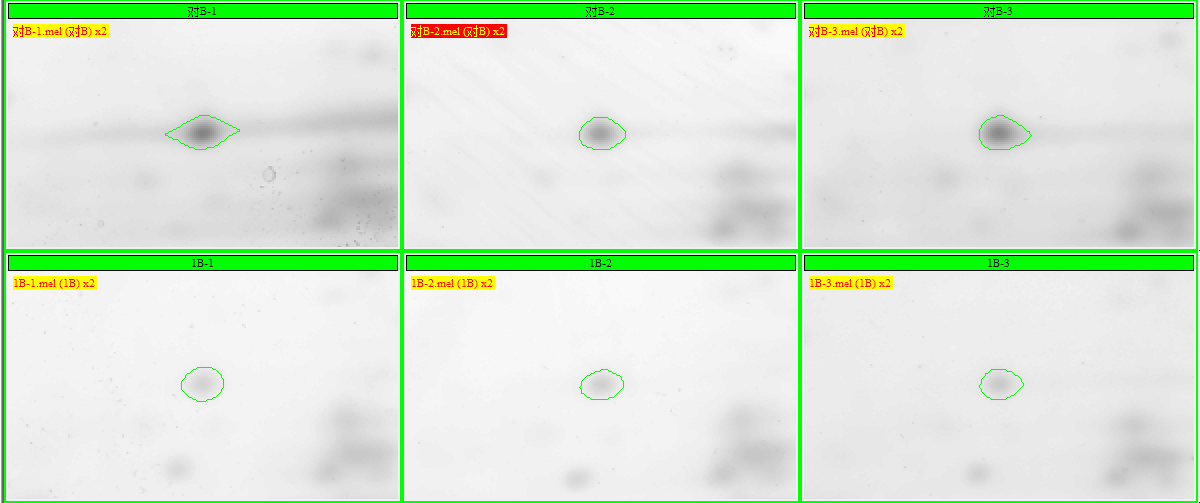
**

**A13**

**
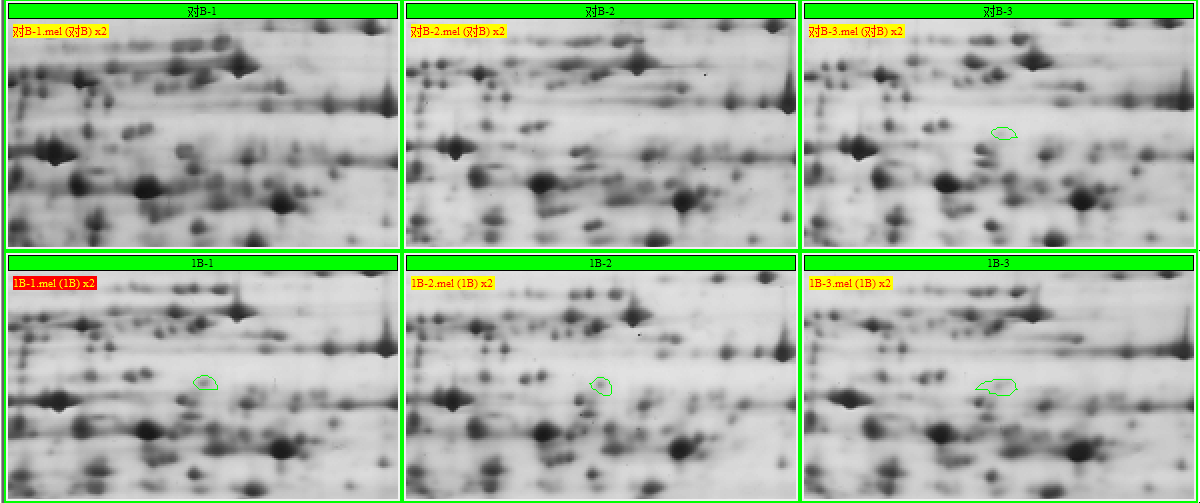
**

**B01**

**
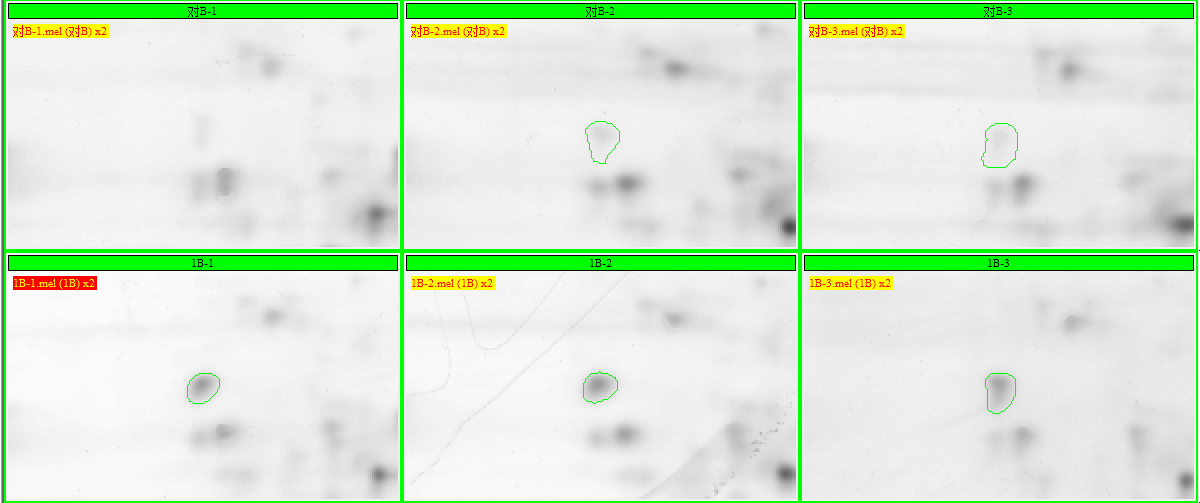
**

**B07**

**
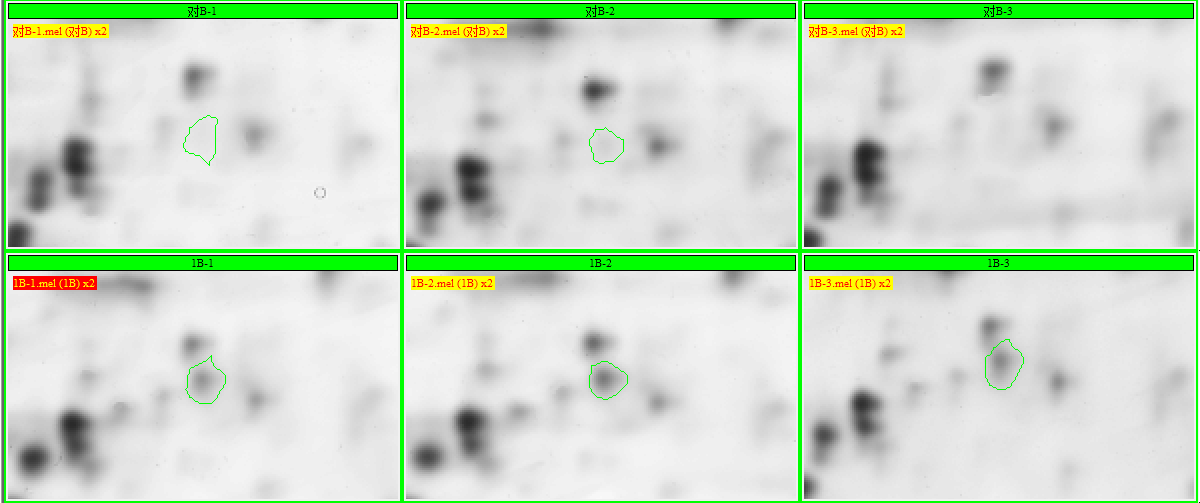
**

**B13**

**
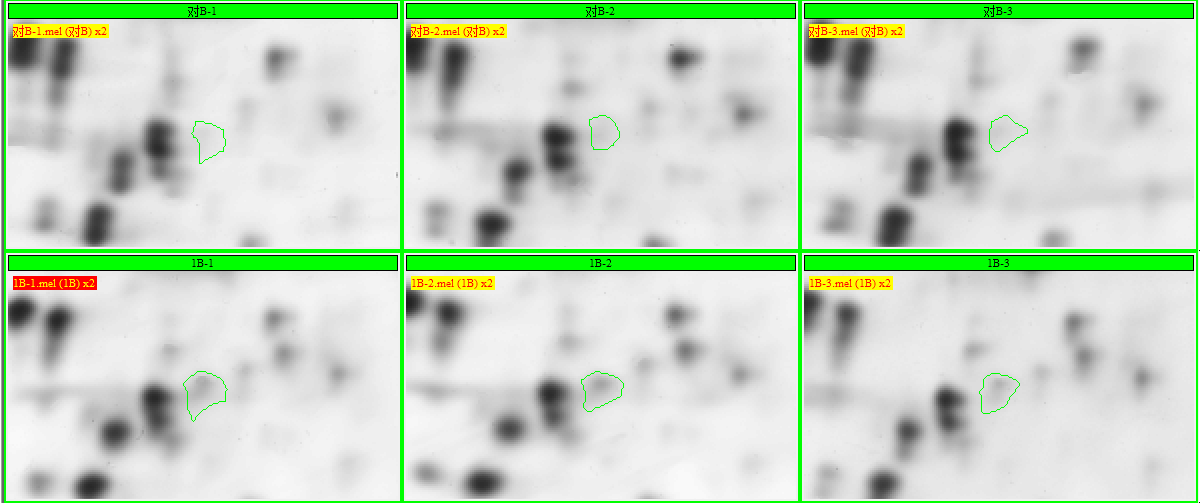
**

**B14**

**
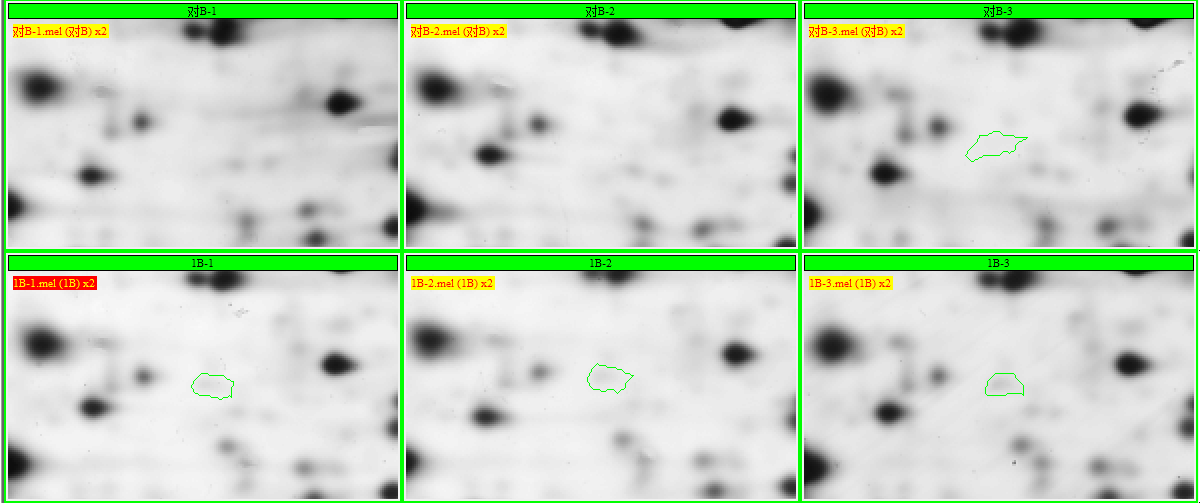
**

**B15**

**
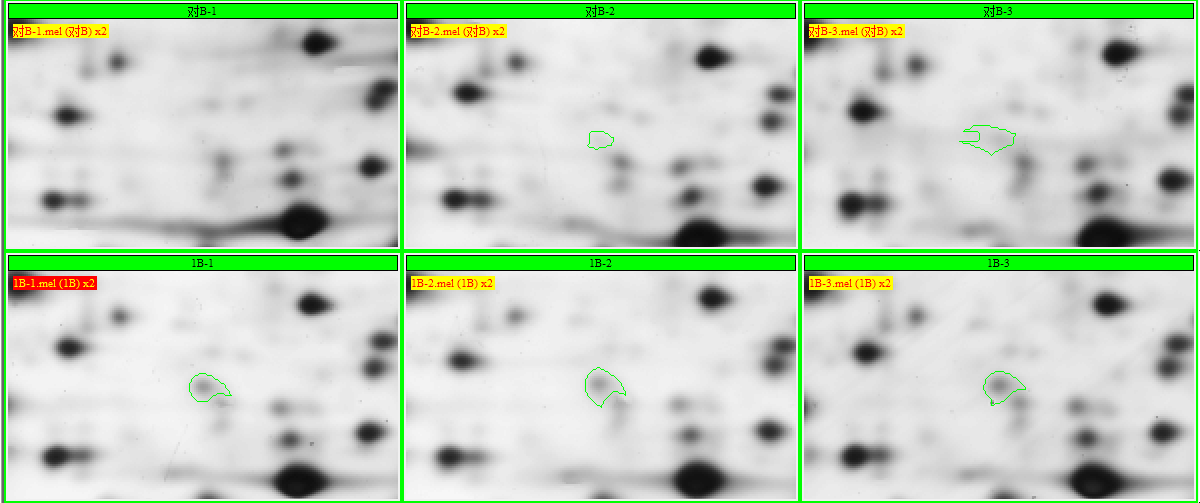
**

**B19**

**
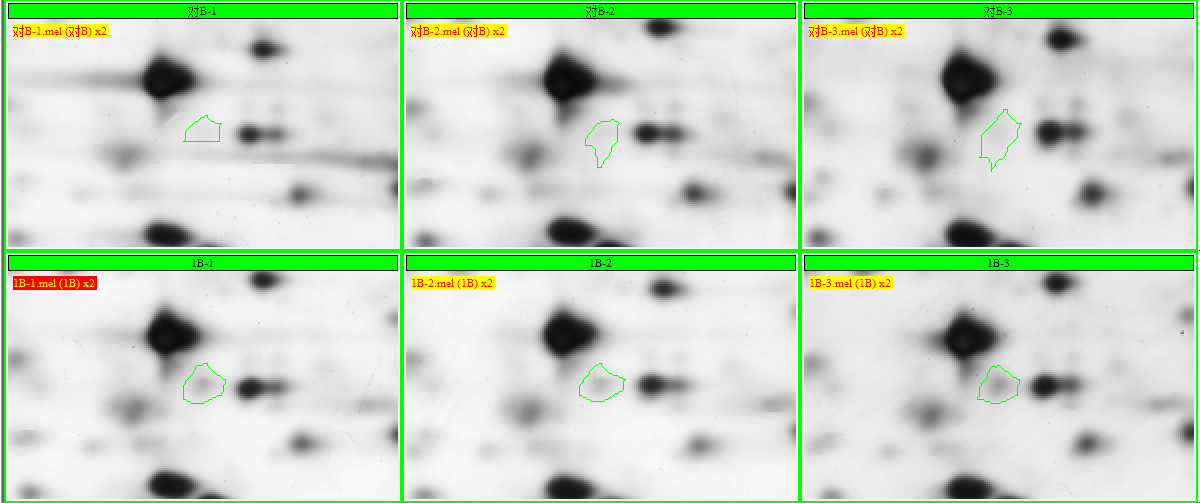
**

**B20**

**
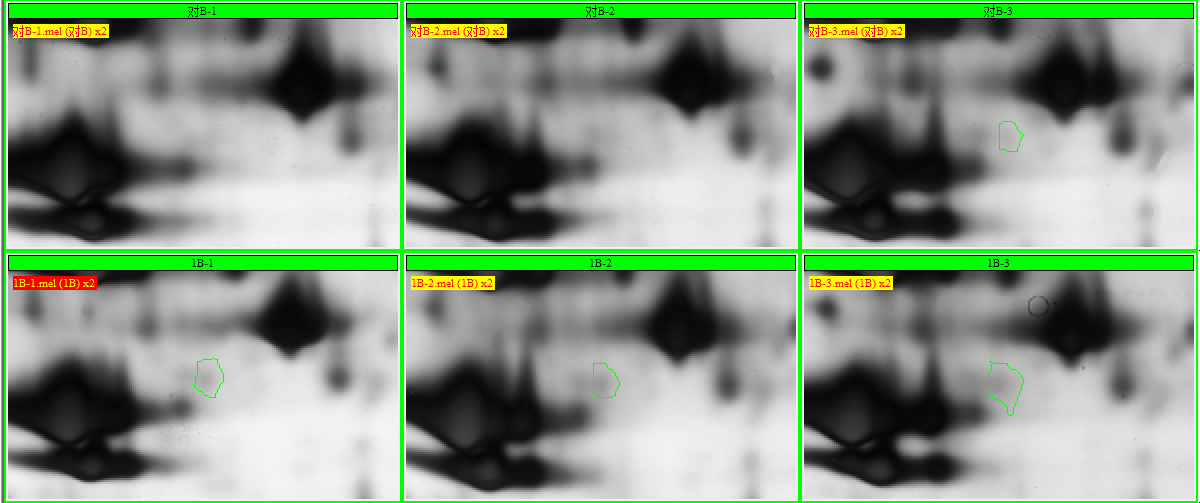
**

**B21**

**
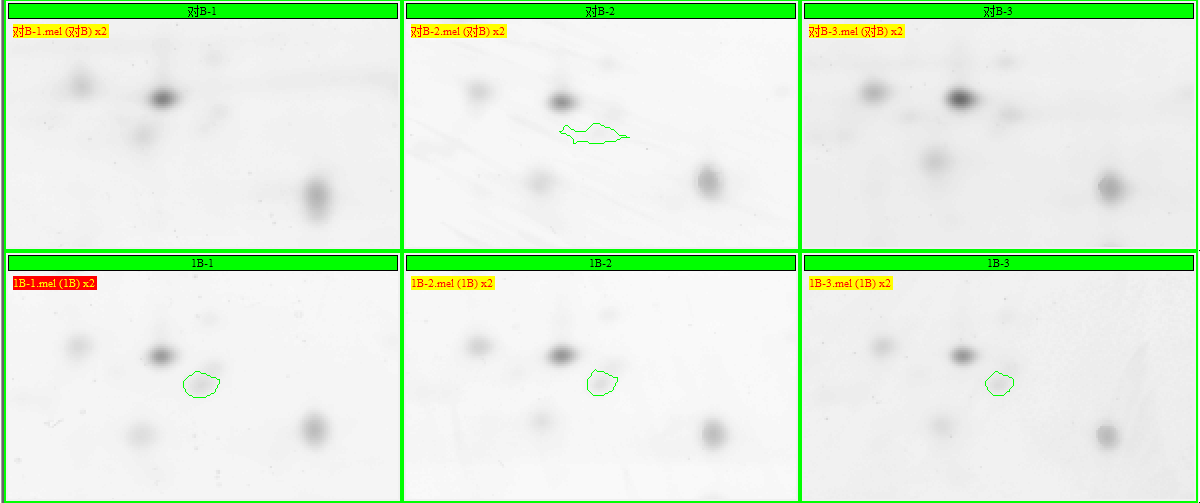
**

**B22**

**
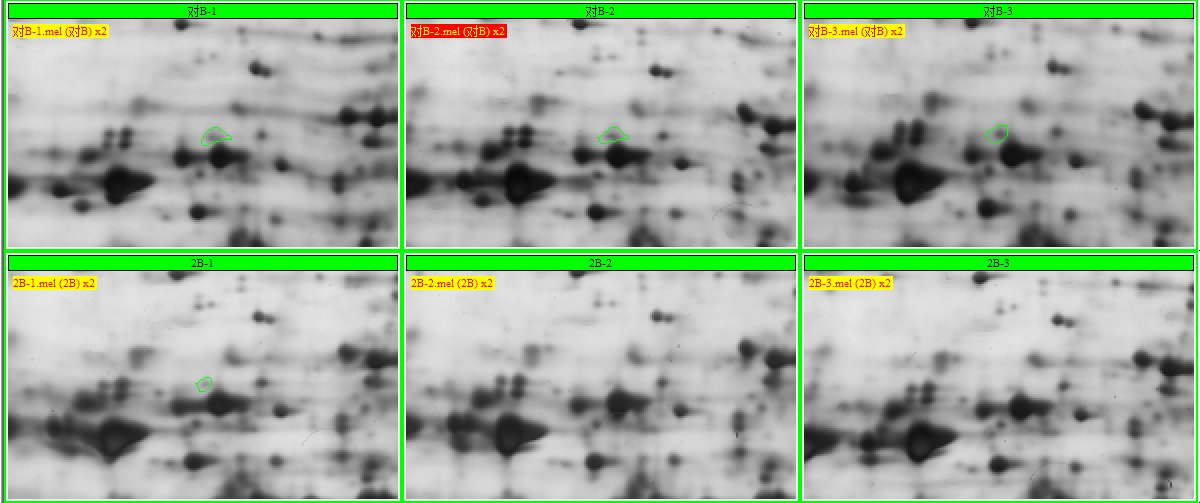
**

**C06**

**
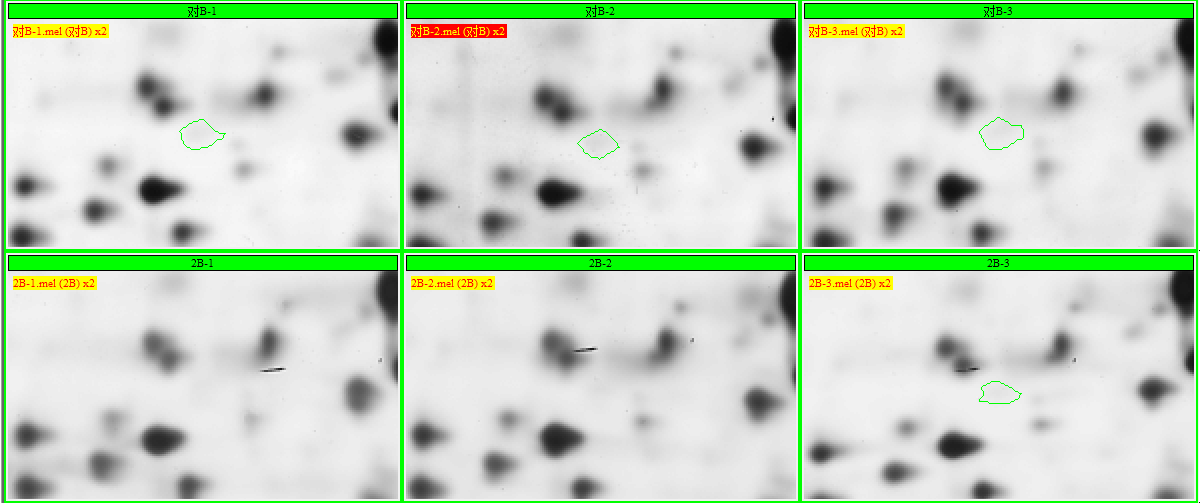
**

**C12**

**
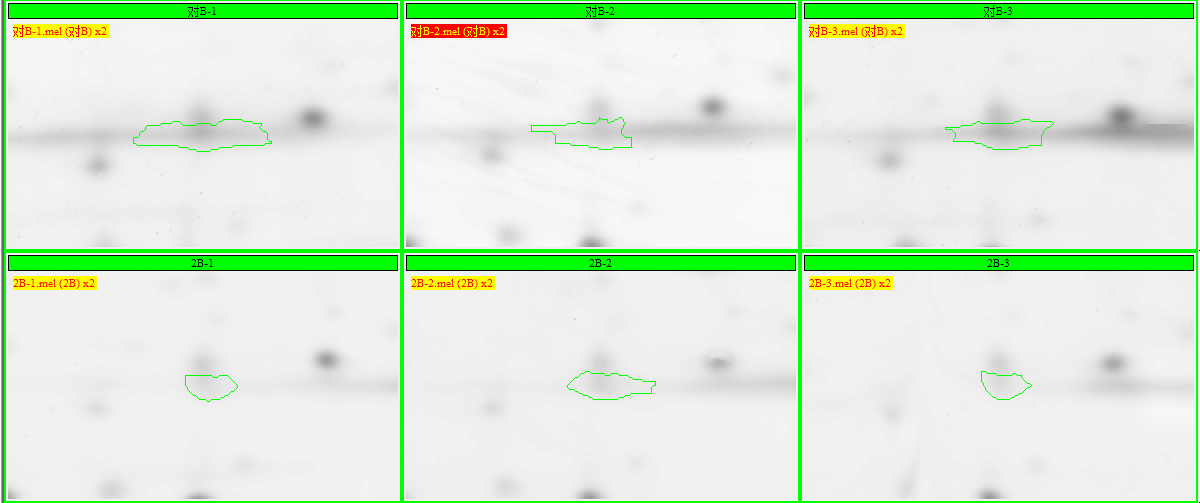
**

**C16**

**
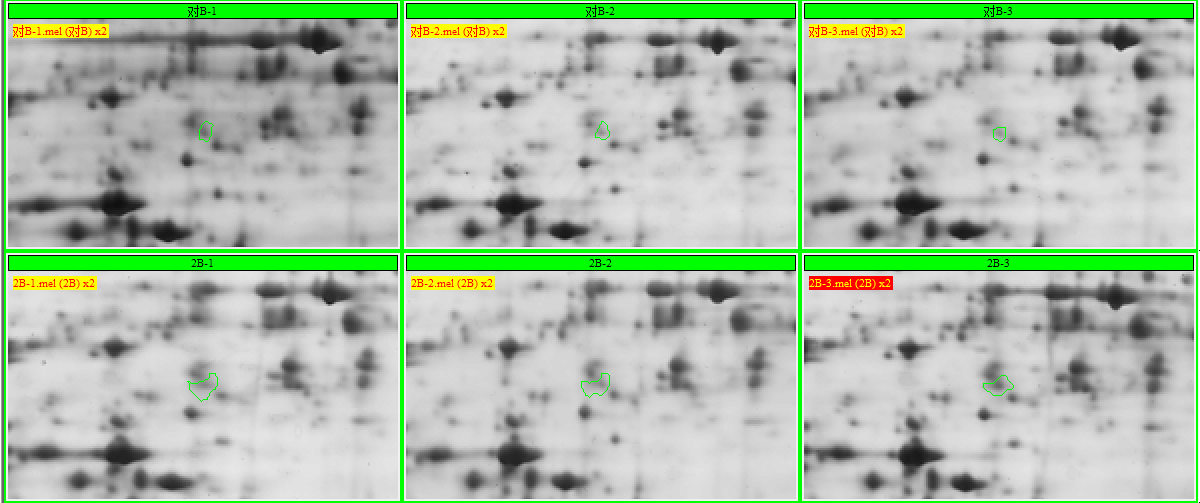
**

**D01**

**
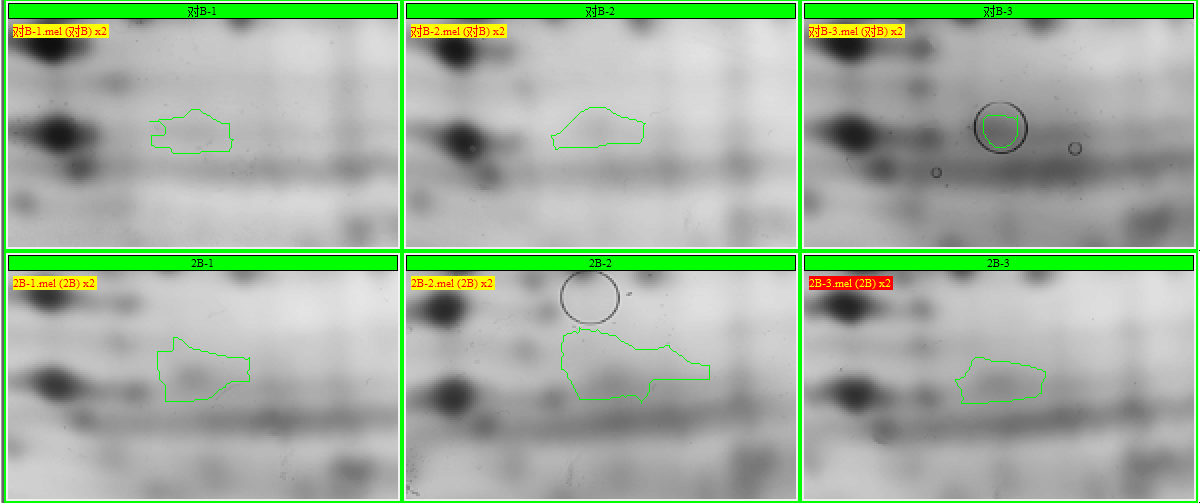
**

**D11**

**
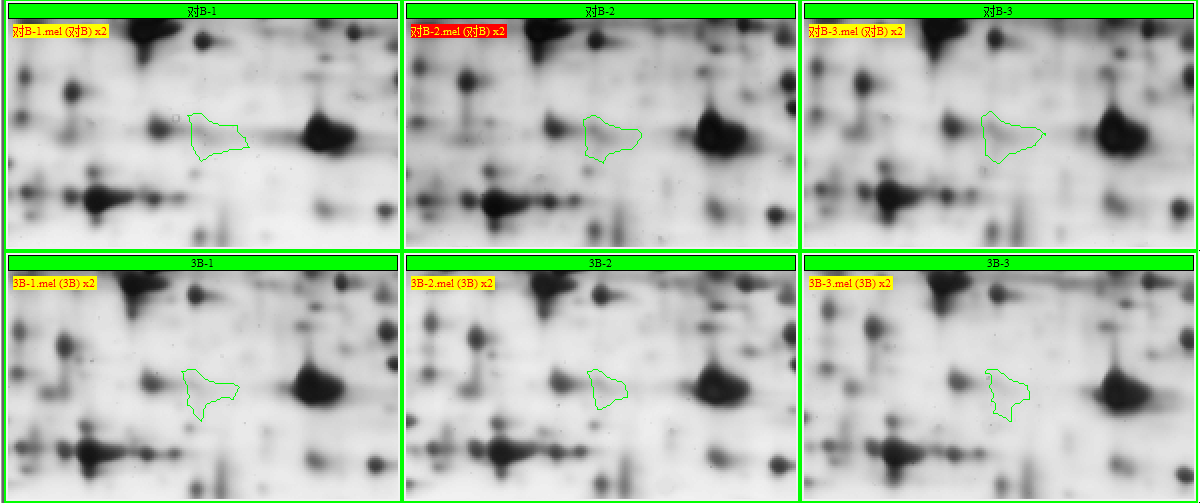
**

**E05**

**
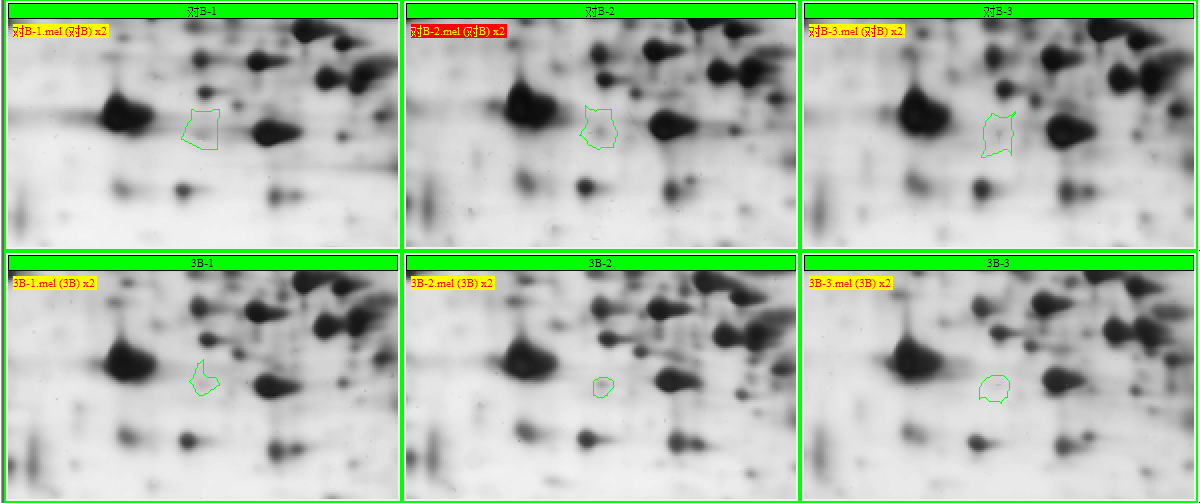
E07**

**
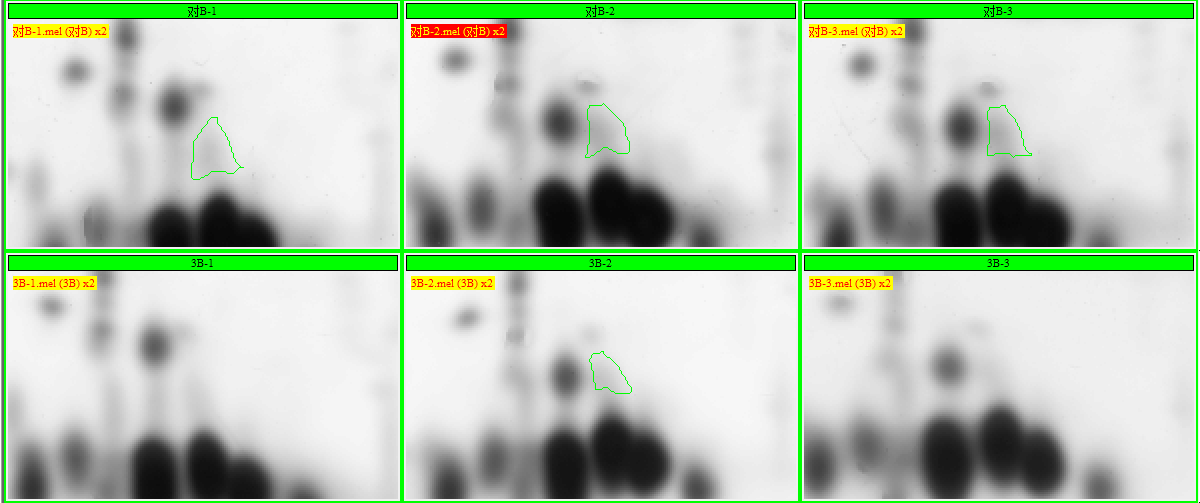
**

**E11**

**
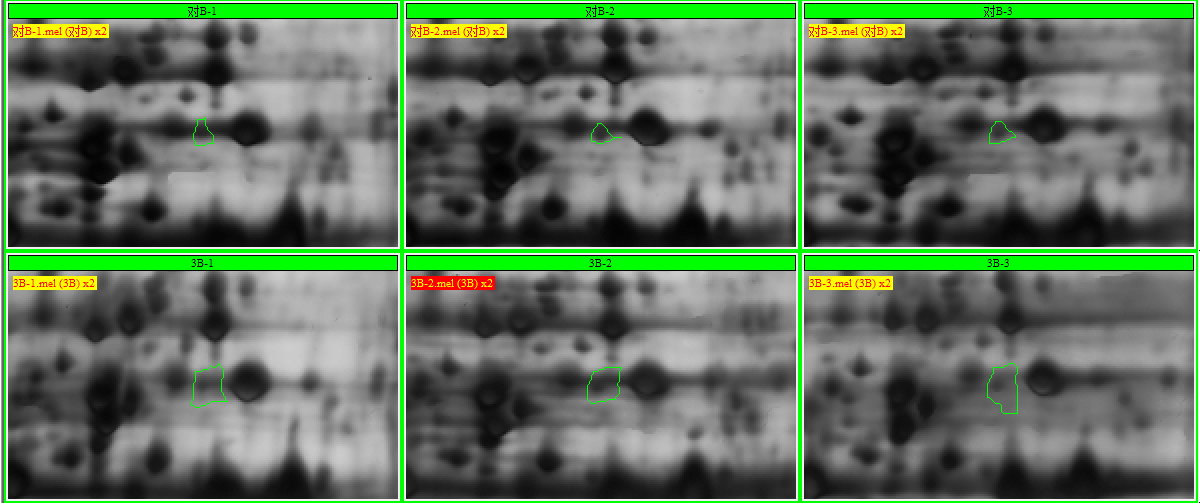
**

**F03**

**
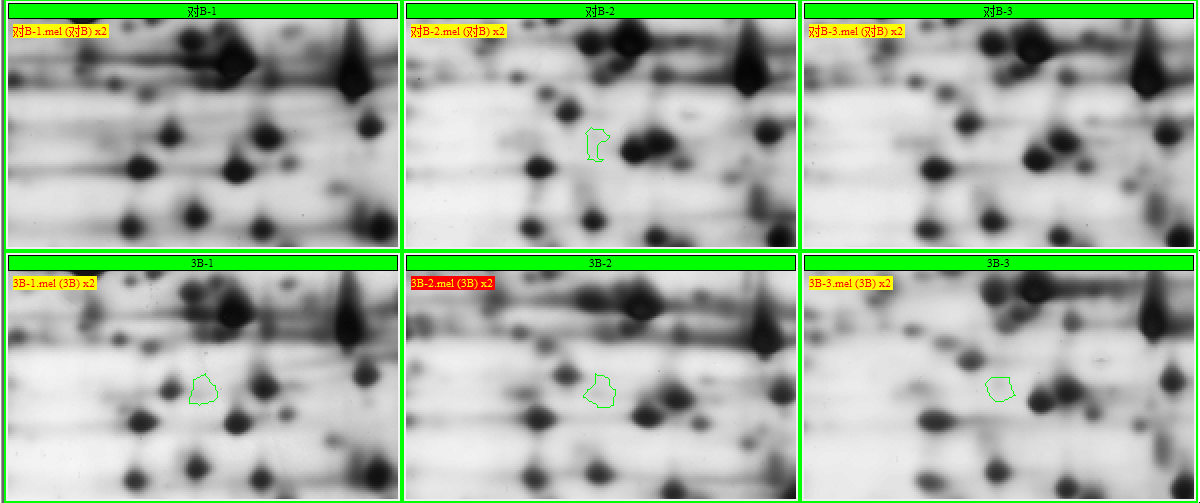
**

**F06**

Supplement: S1 File — (DOCX) [file pone.0182671.s001.docx]

1. **PMF of** **protein spot from** **MALDI-TOF- MS**

**1) A07**


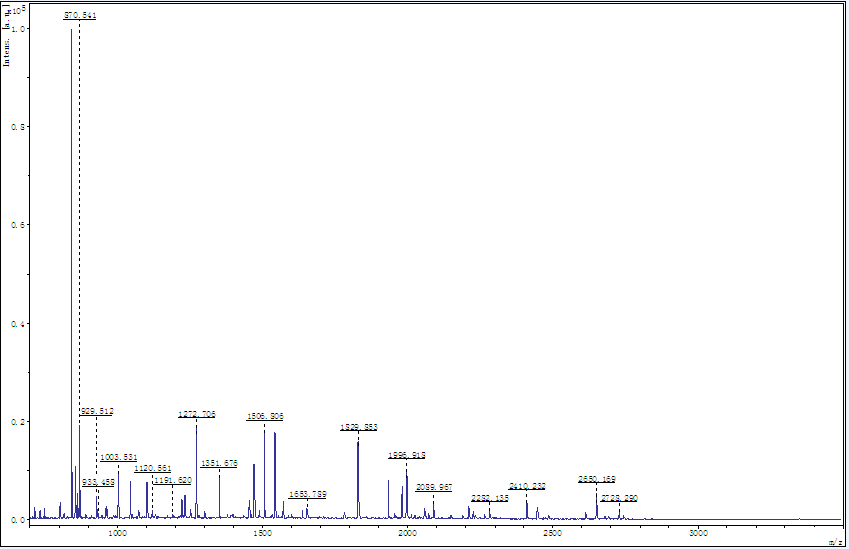


**2) A12**


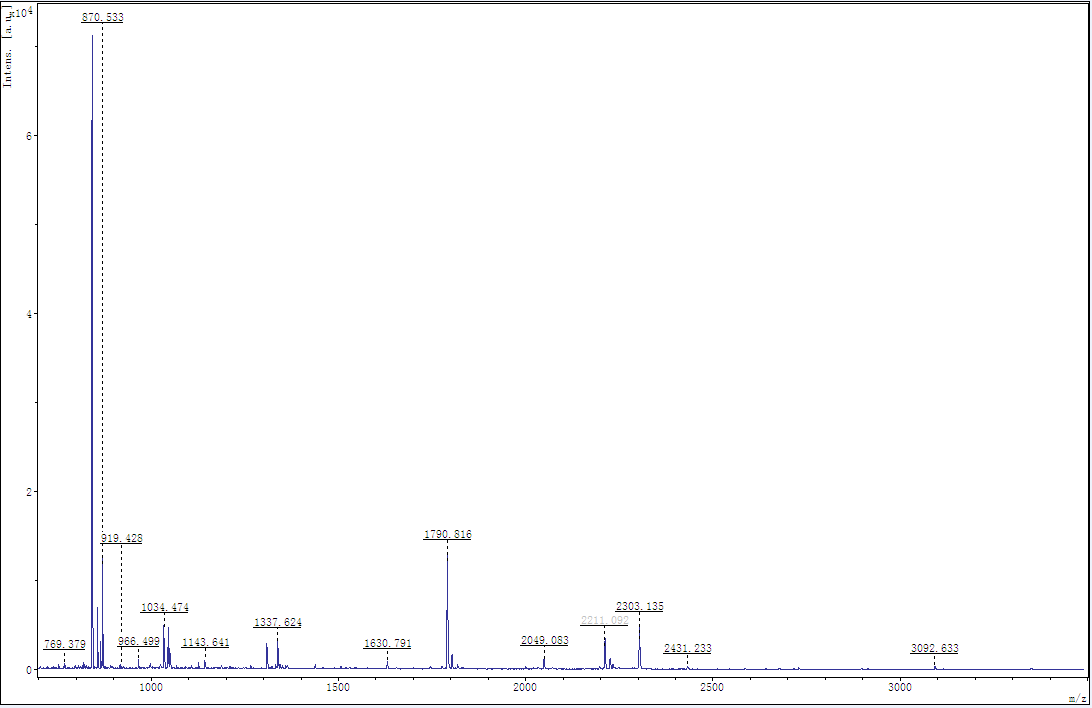


**3) A13**


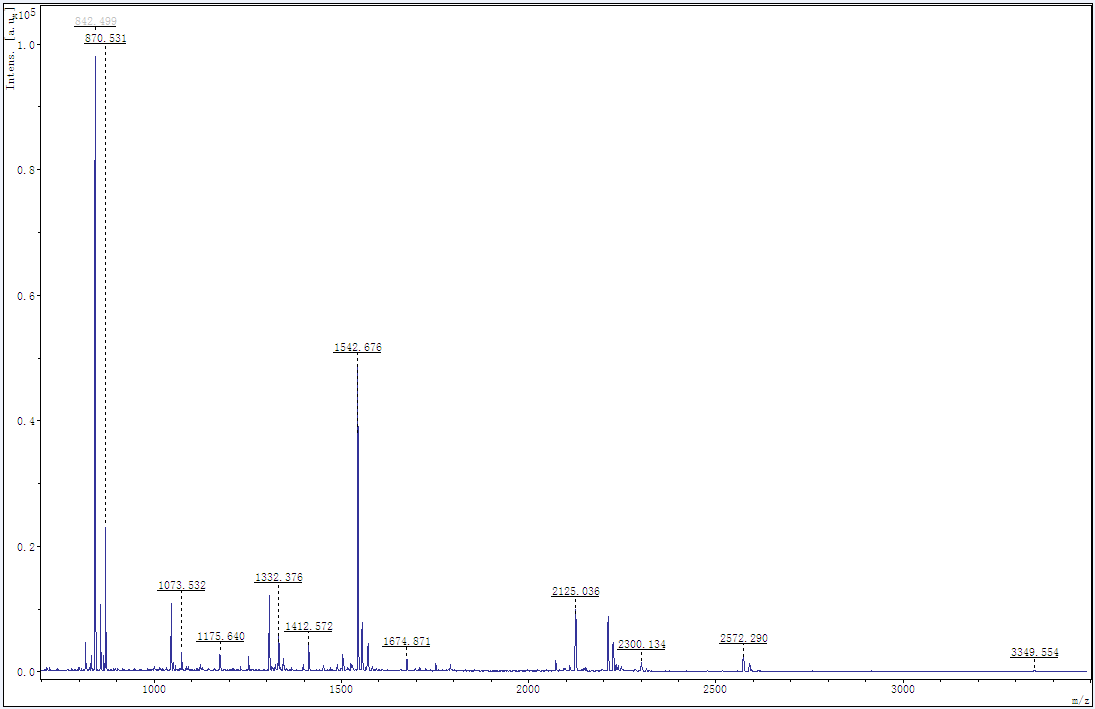


**4) B01**


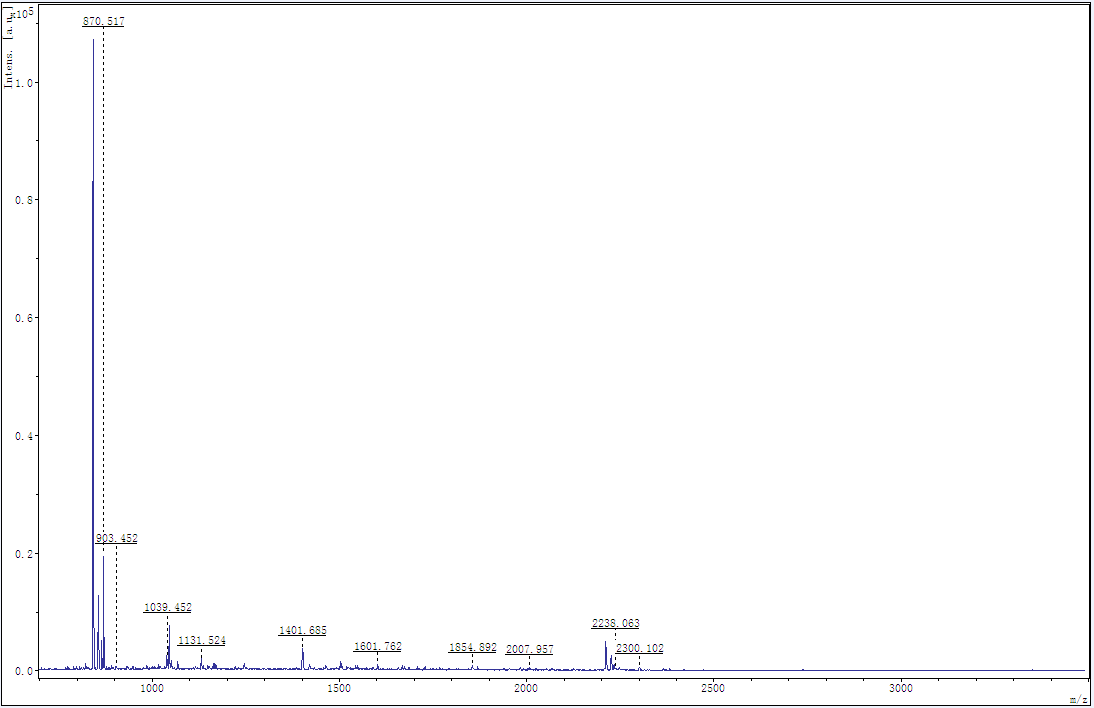


**5) B07**


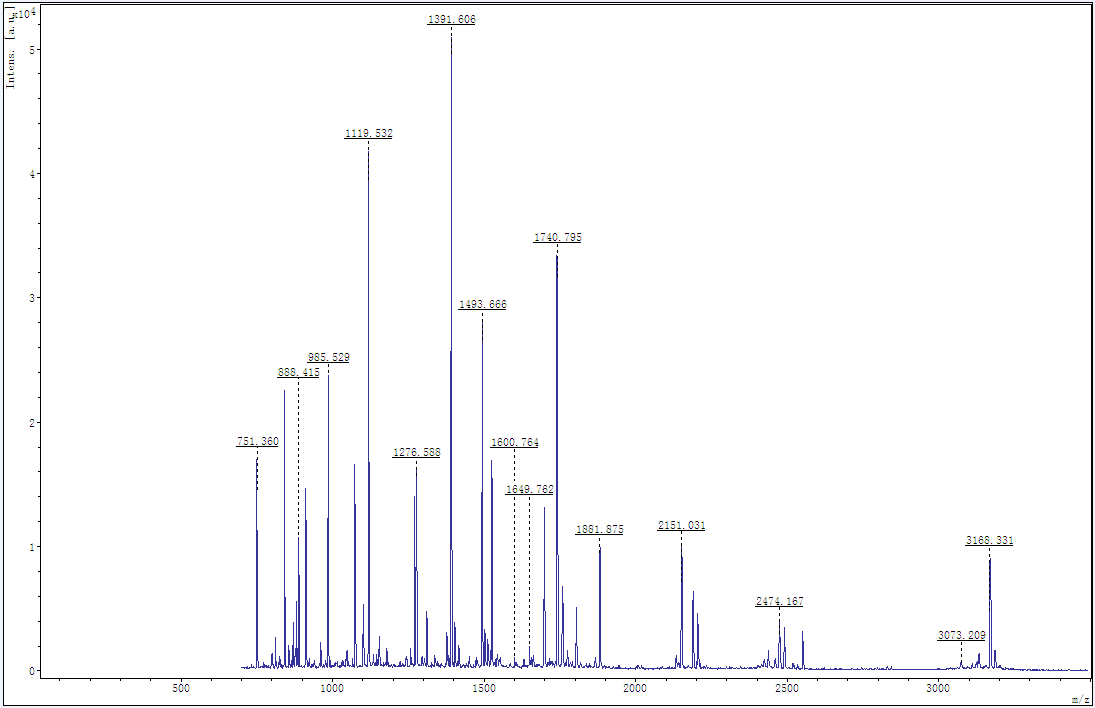


**6) B13**


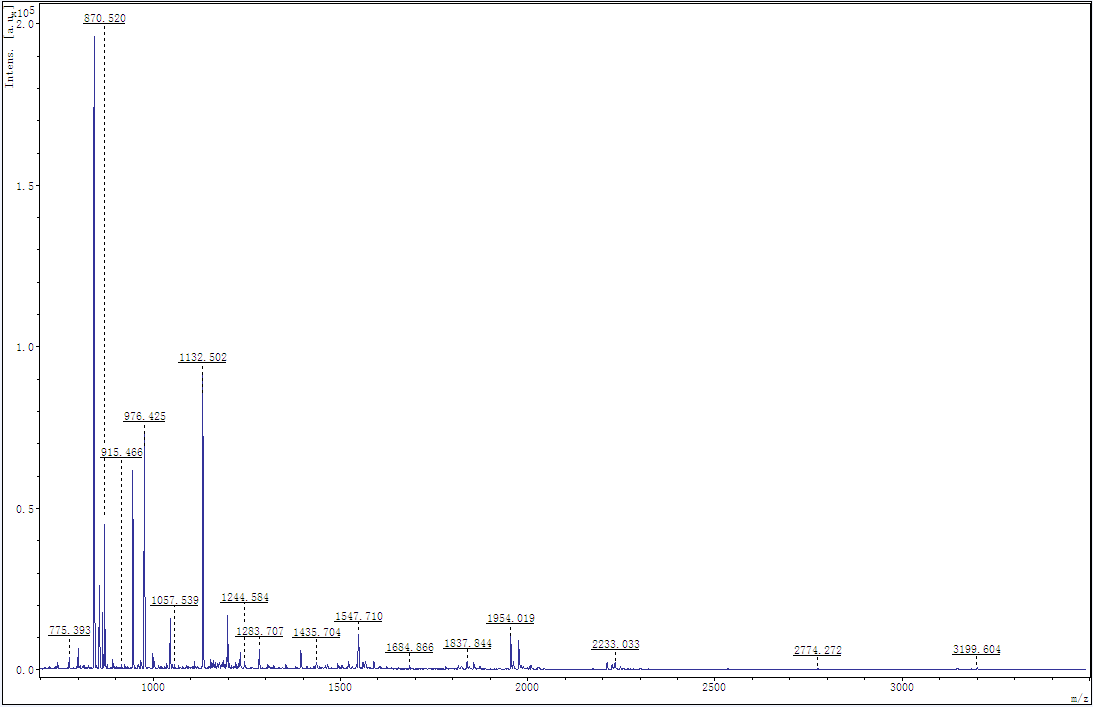


**7) B14**


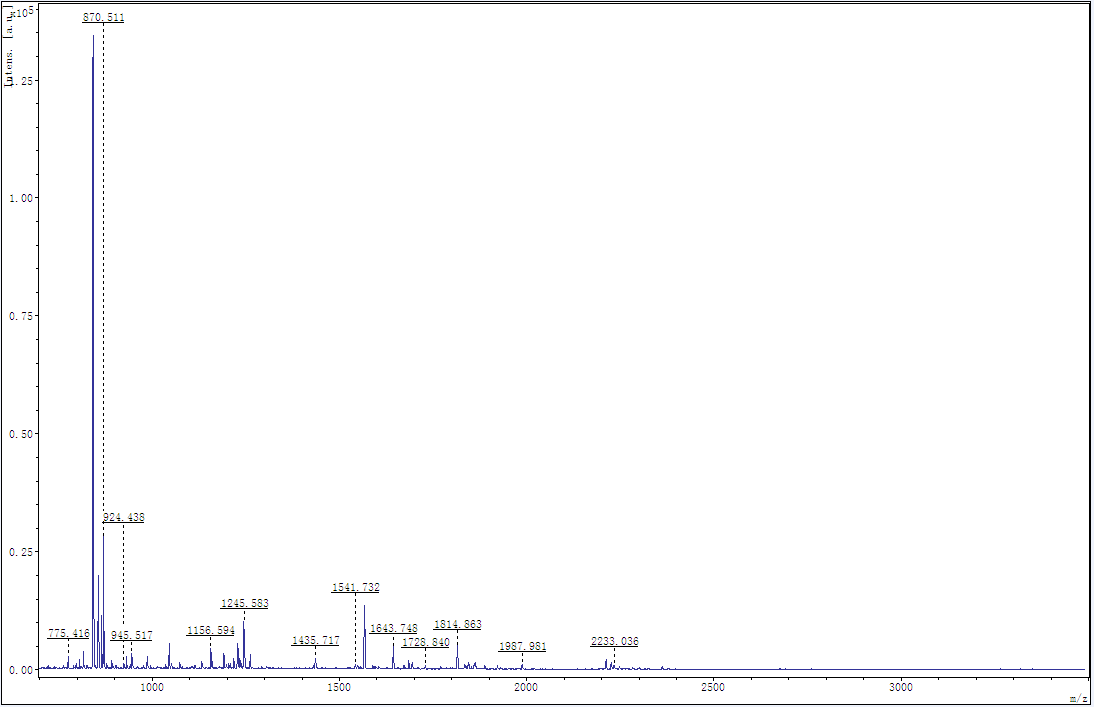


**8) B15**


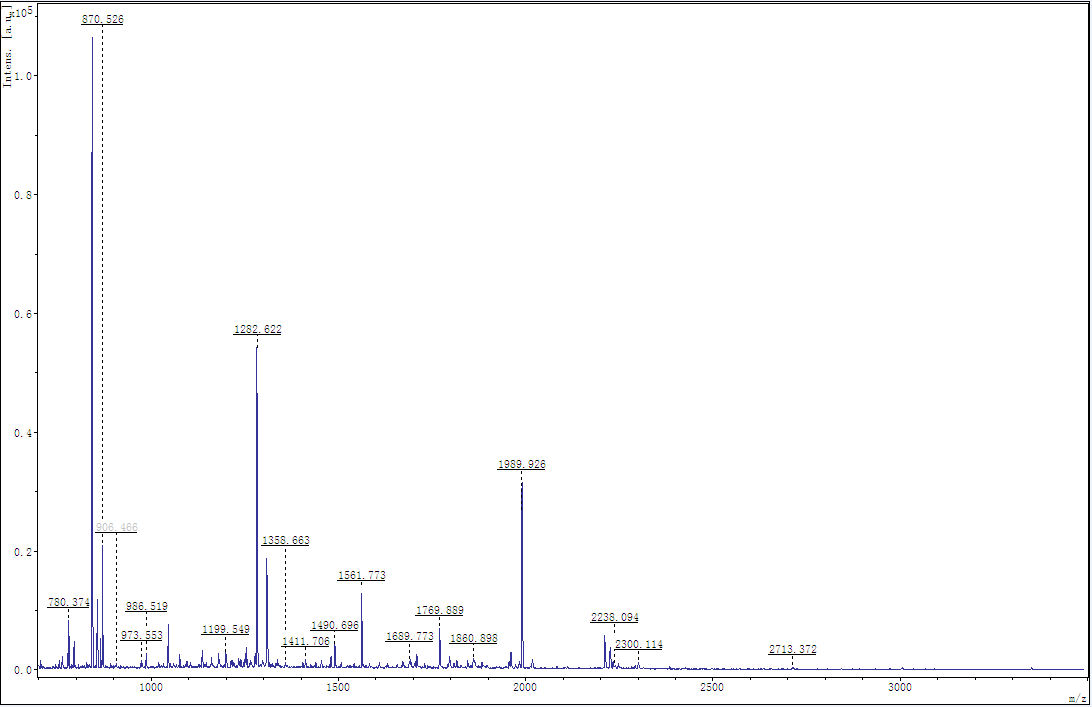


**9) B19**


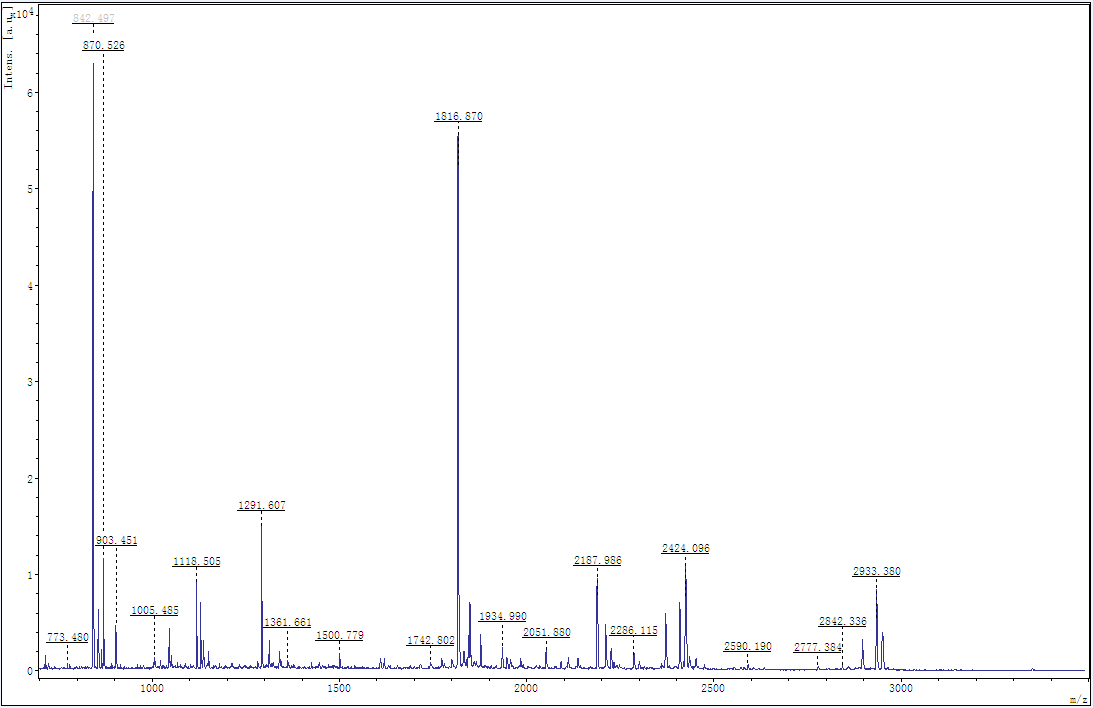


**10) B20**


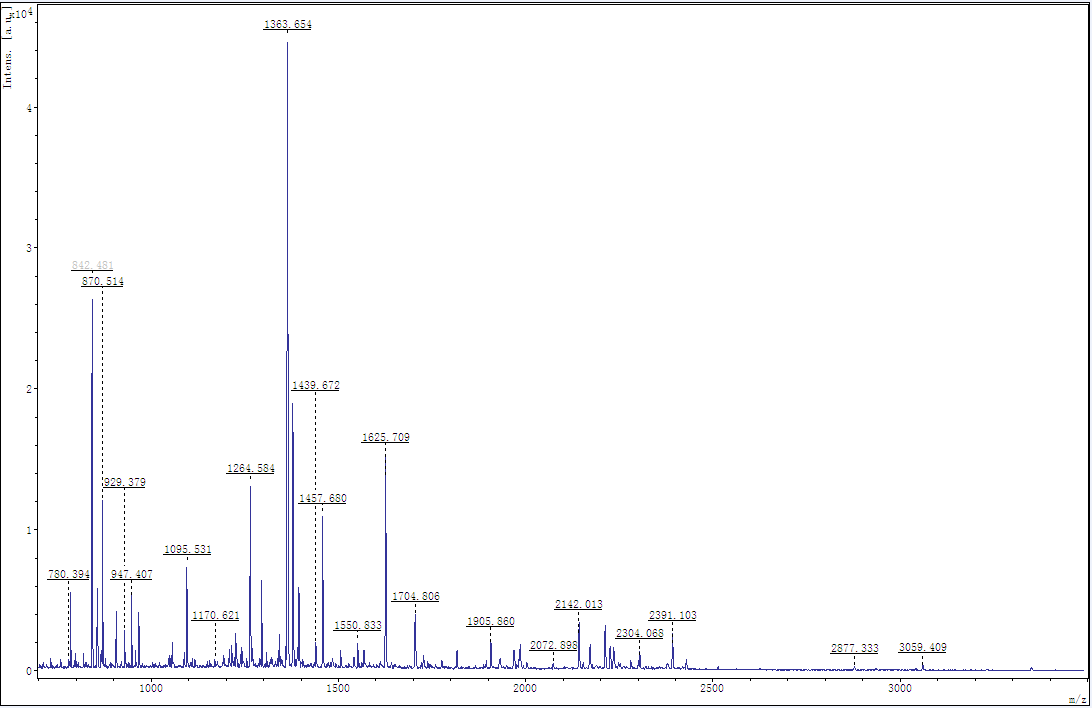


**11) B21**


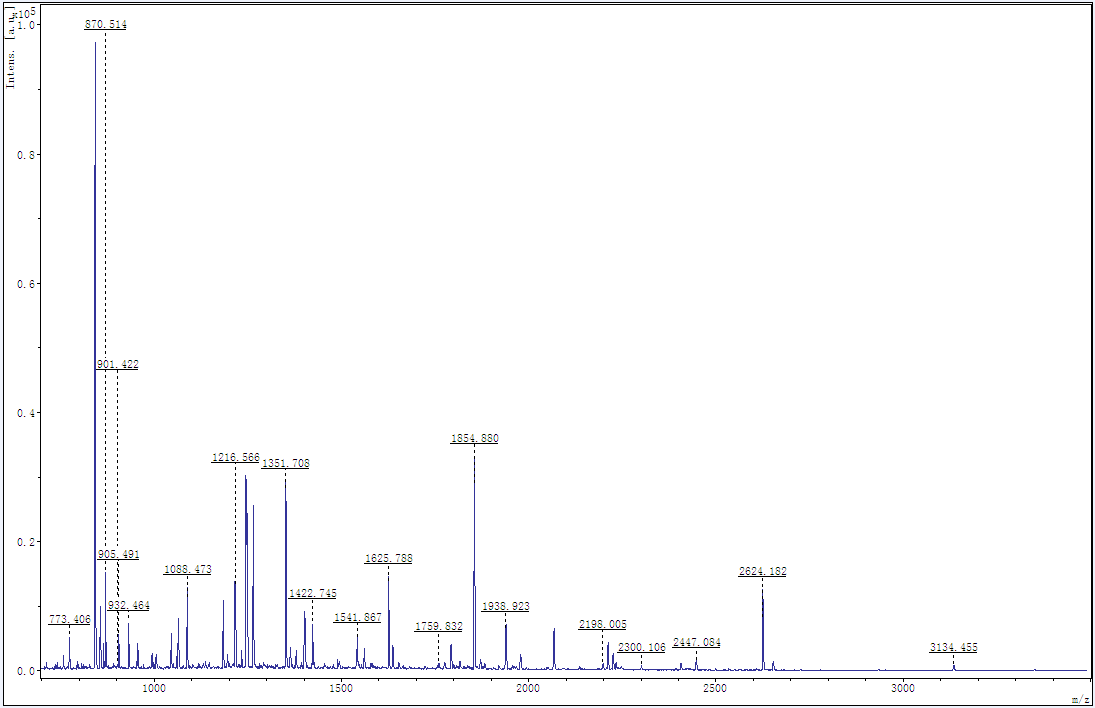


**12) B22**


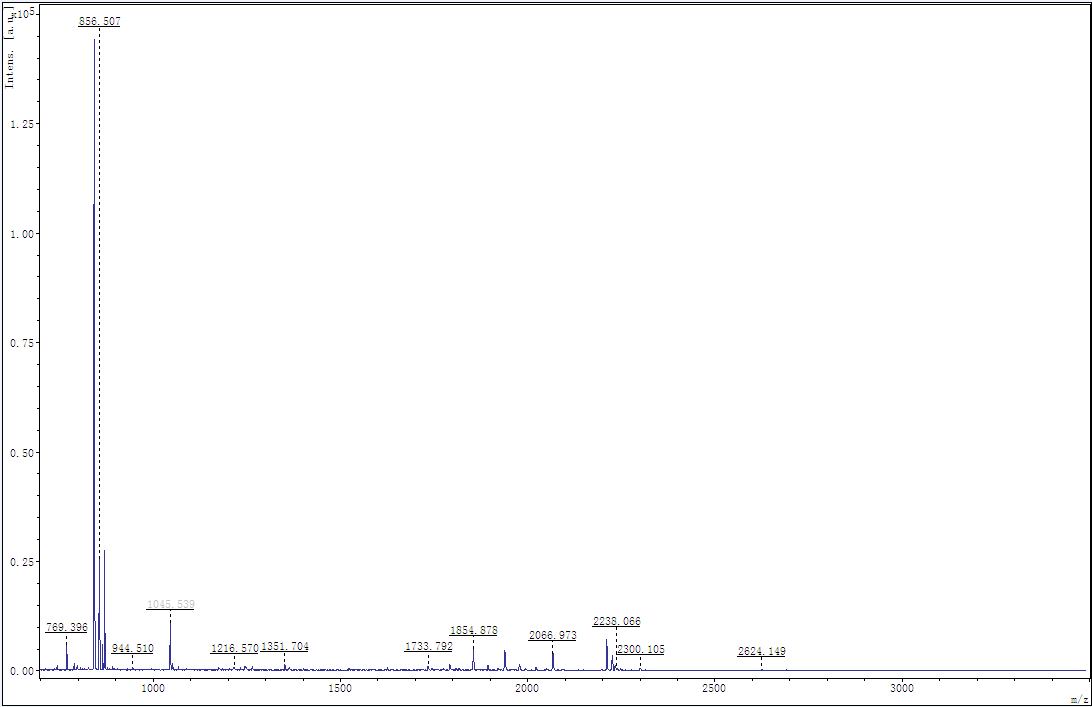


**13) C06**


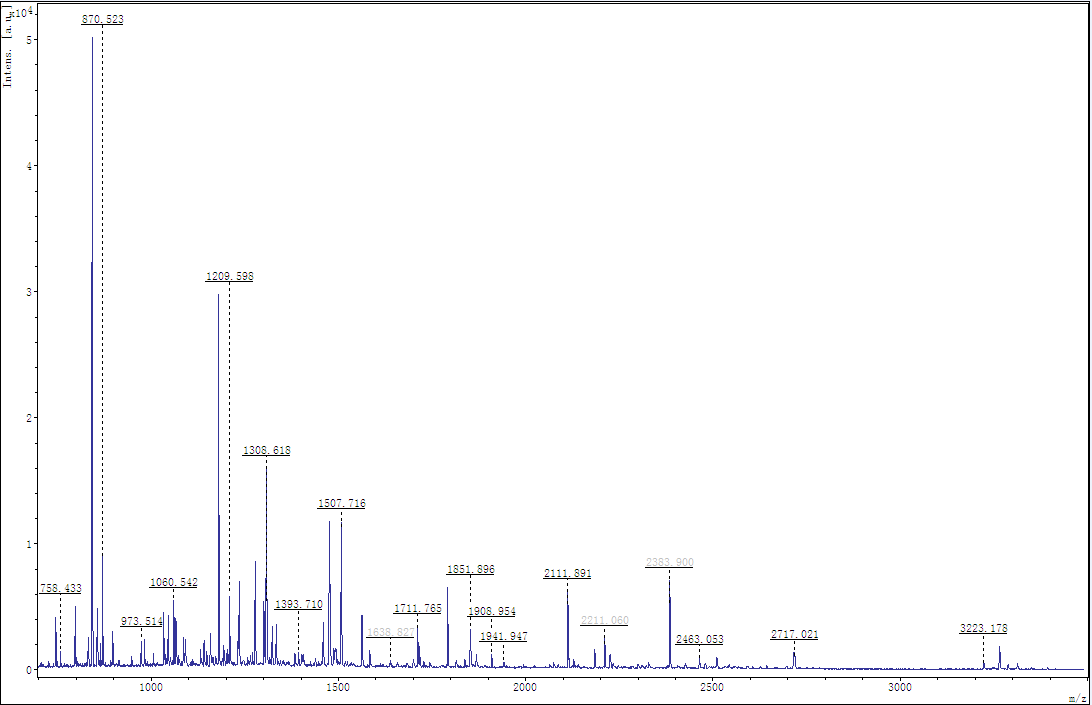


**14) C12**


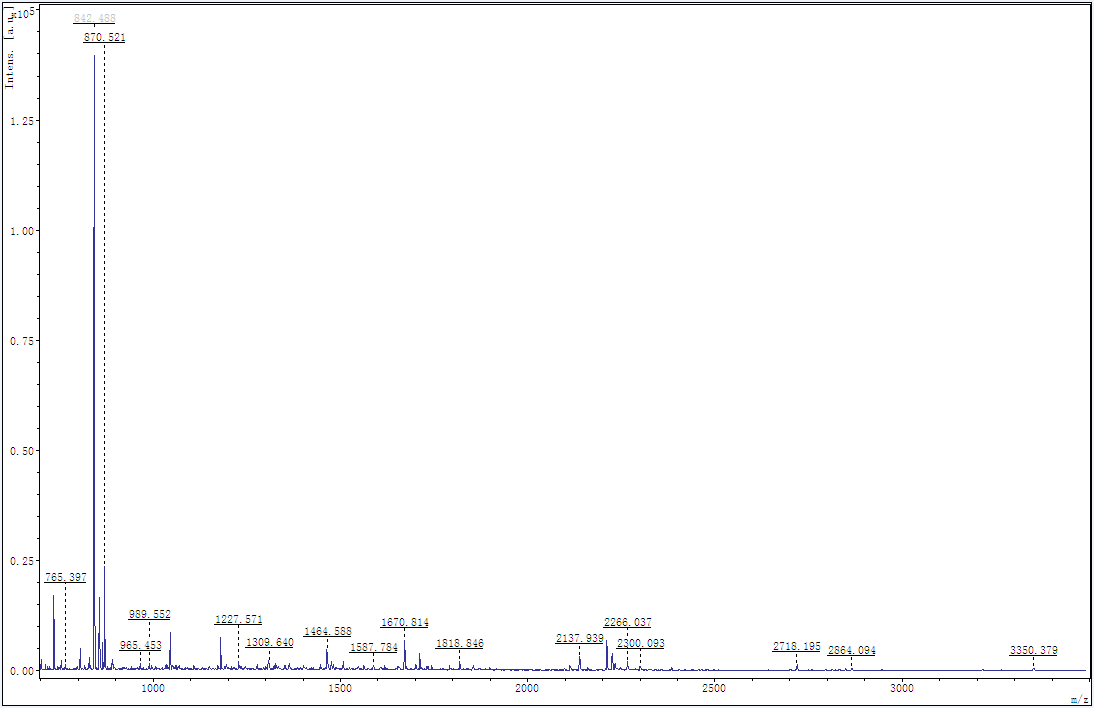


**15) C16**


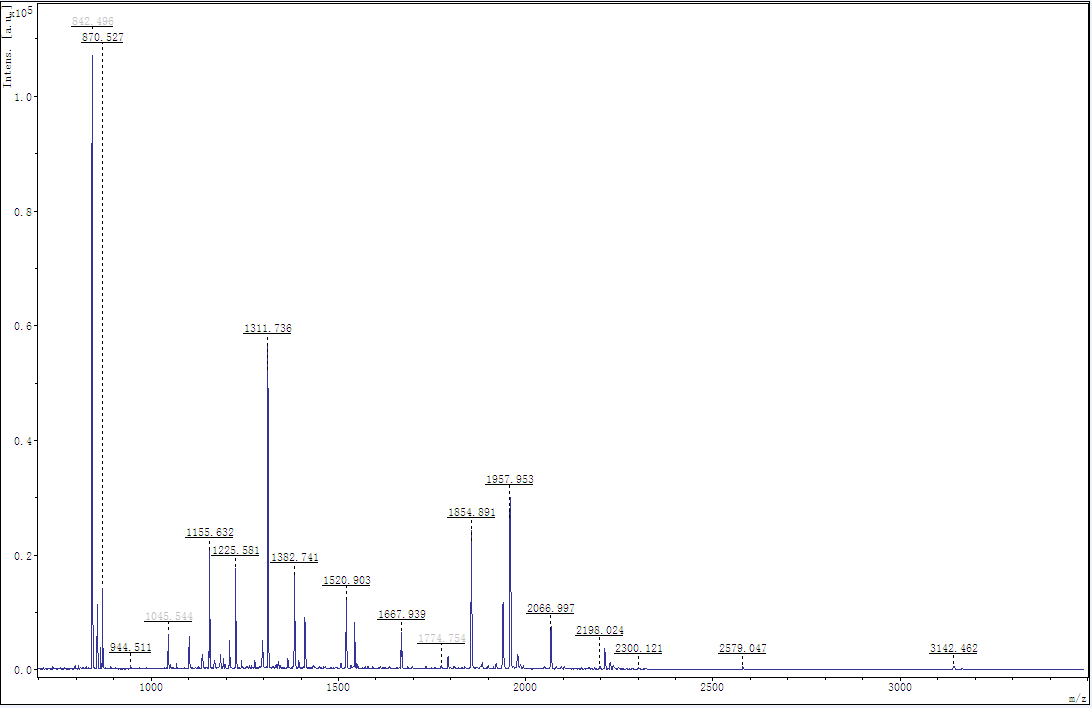


**16) D01**


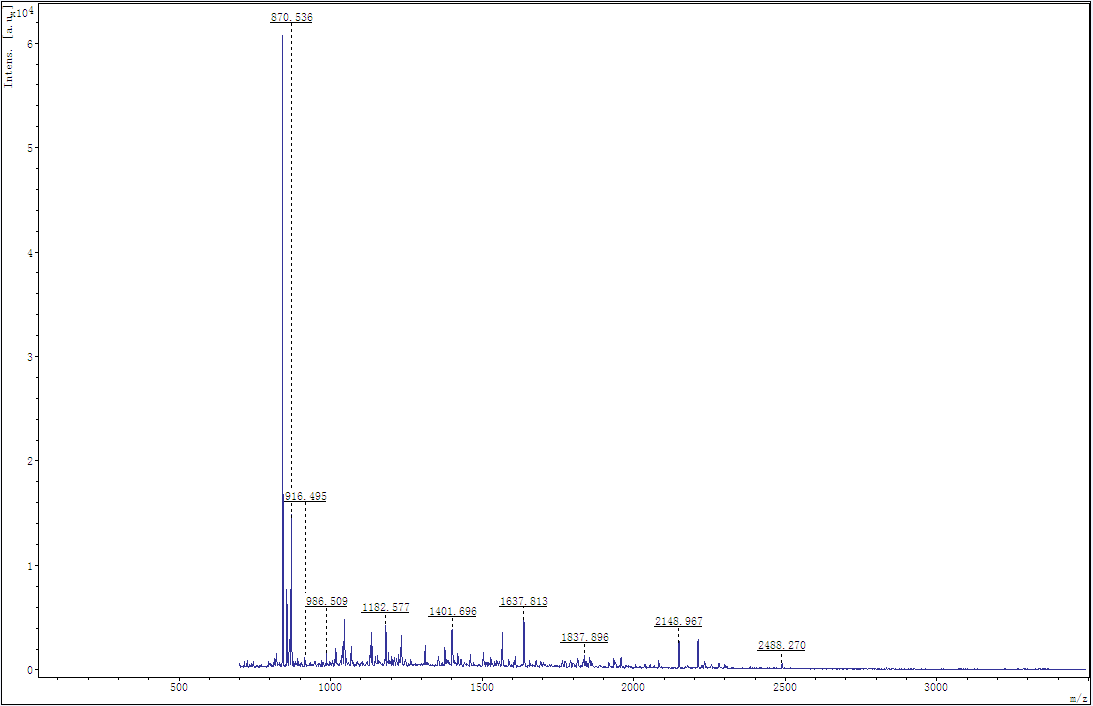


**17) D11**


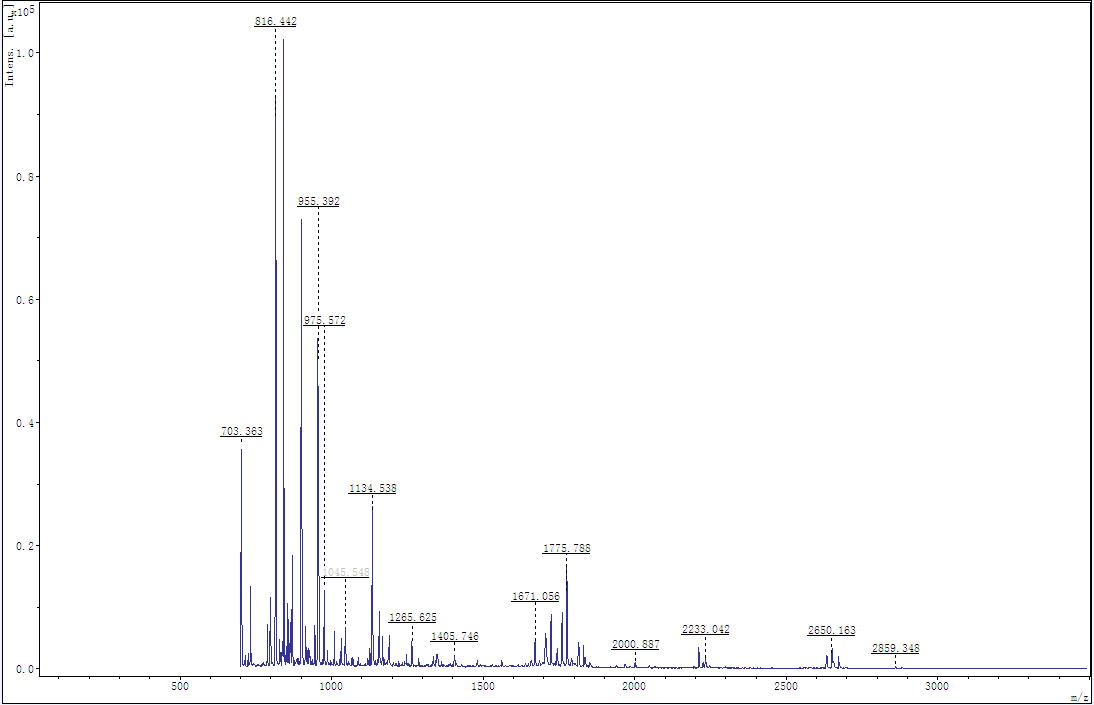


**18) E05**


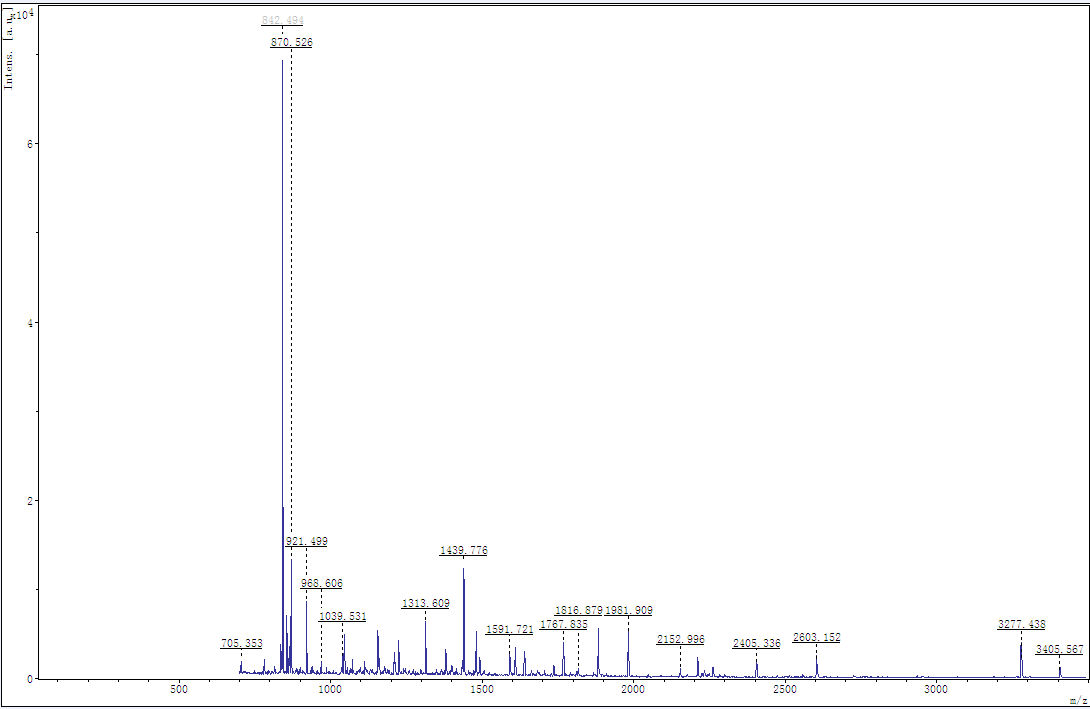


**19) E07**


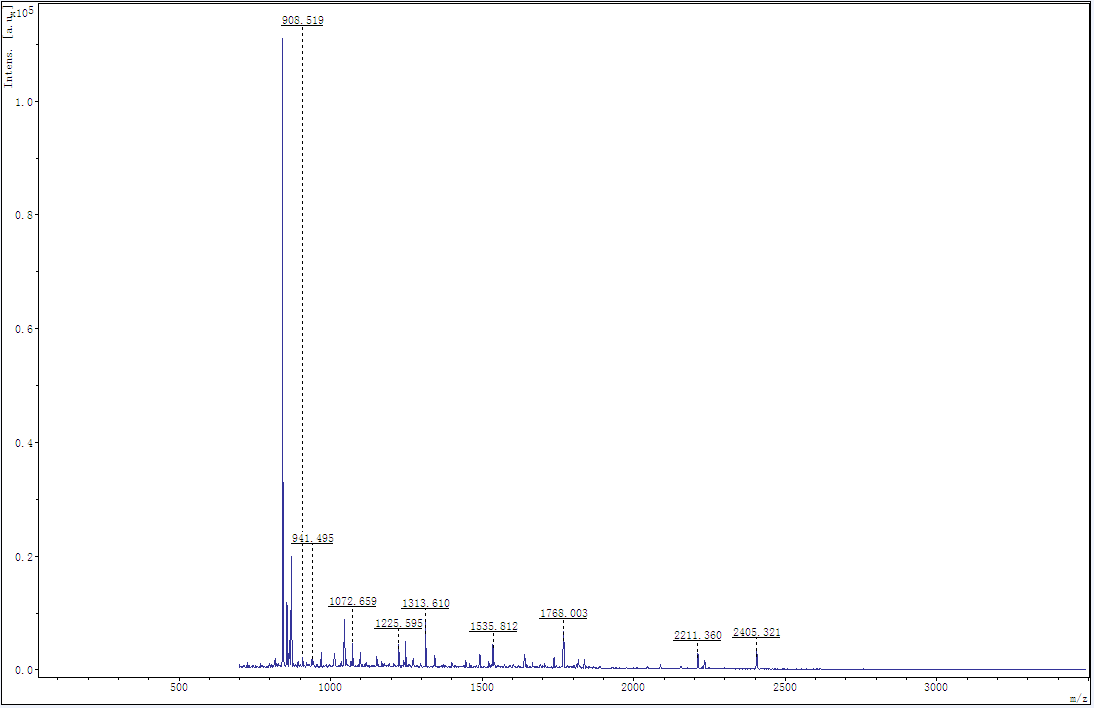


**20) E11**


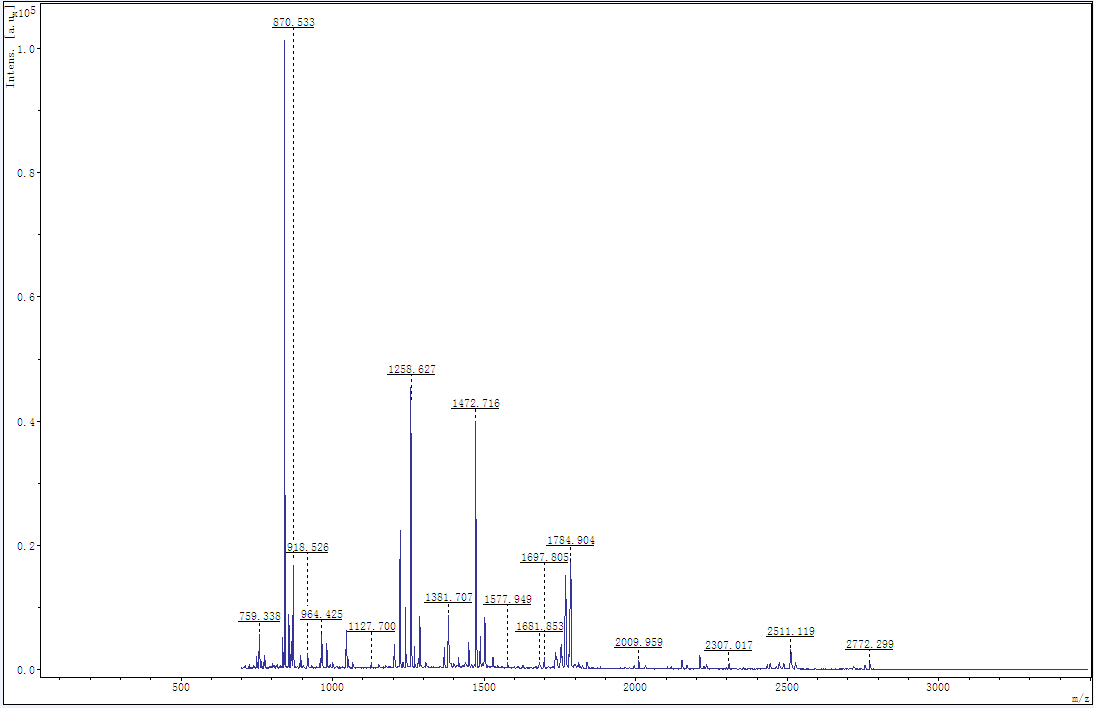


**21) F03**


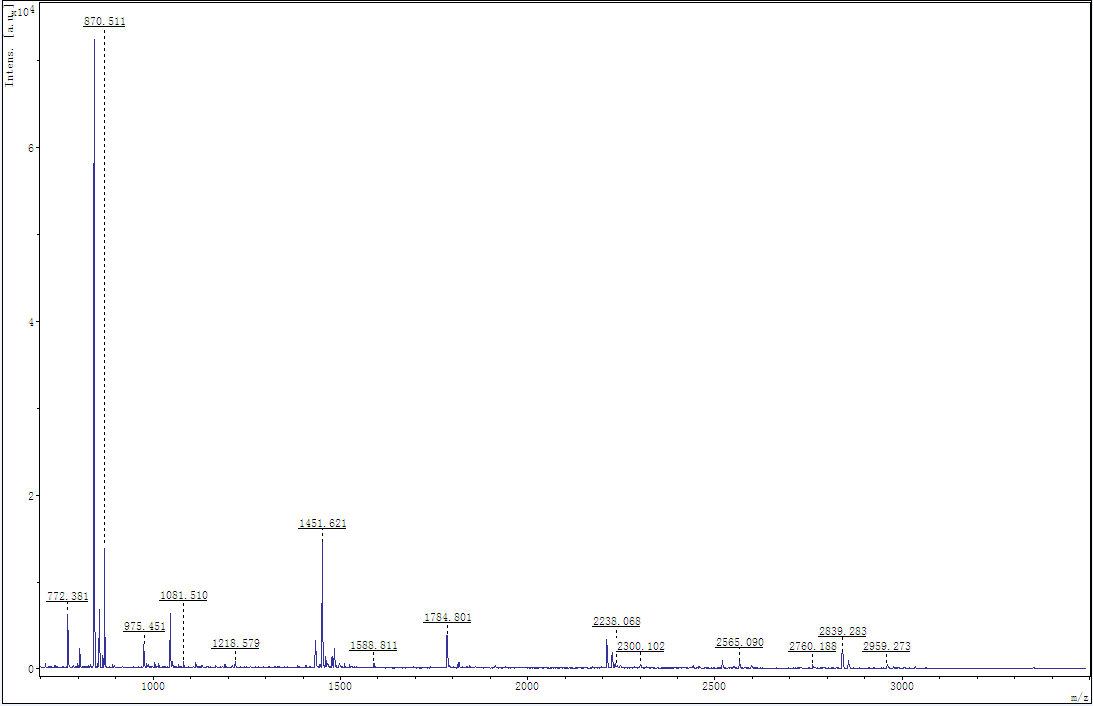


**22) F06**


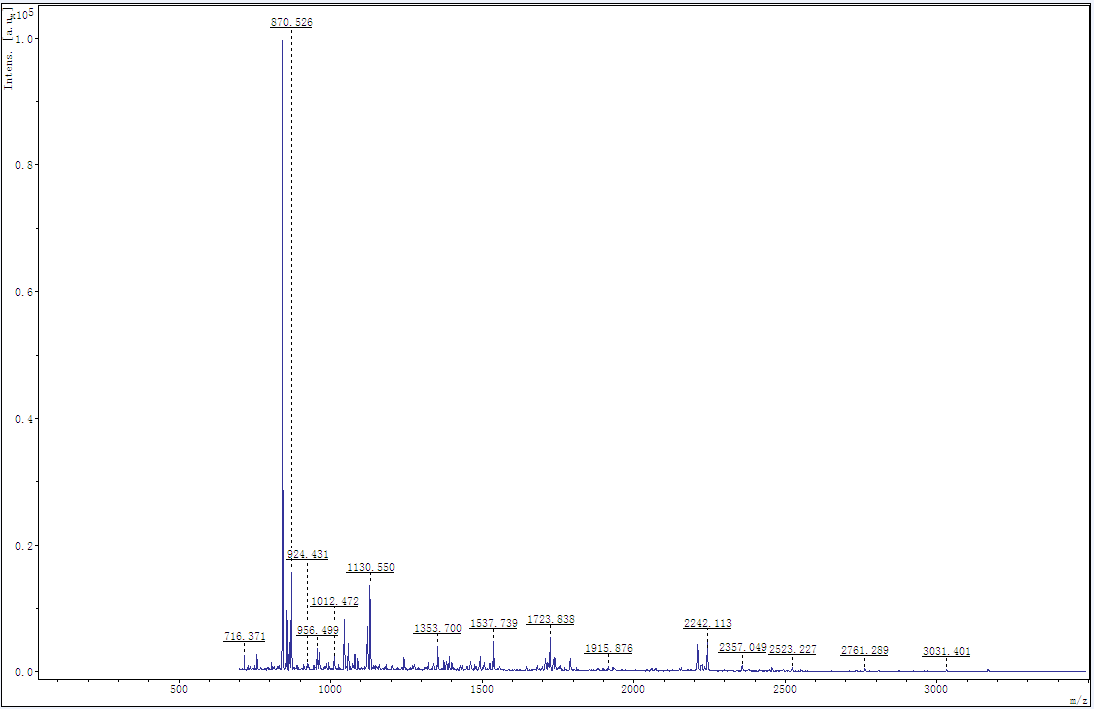

Supplement: S2 File — (DOCX) [file pone.0182671.s002.docx]
