# Supplementary material for: Up-regulation of calreticulin in mouse liver tissues after long-term irradiation with low-dose-rate gamma rays
Source: PLoS One. 2017 Sep 20;12(9):e0182671. doi: 10.1371/journal.pone.0182671 (PMC5607120; doi:10.1371/journal.pone.0182671)
Supplement: S3 File — (DOCX) [file pone.0182671.s003.docx]

**Real time PCR:**

1. **Amplification Plot**


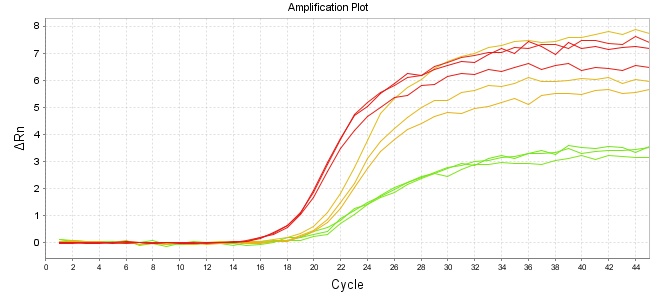


**(1) mus-GAPDH-Amplification Plot**


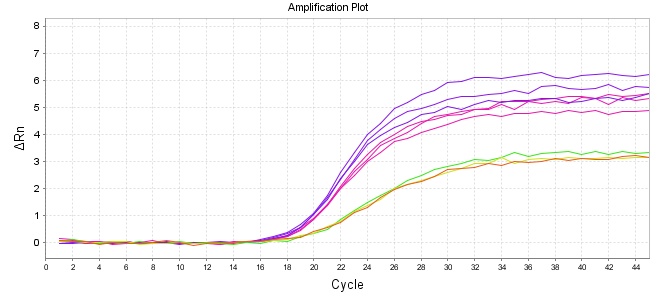


(2) cat-Amplification Plot


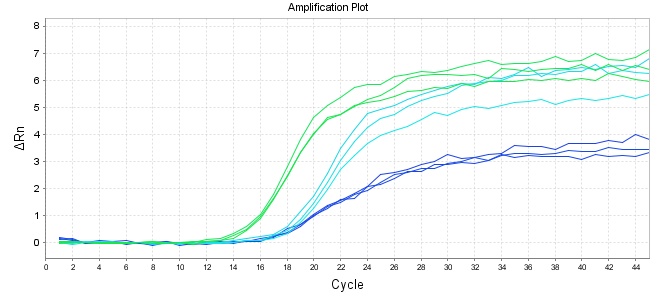


1. GSTP1-Amplification Plot

**
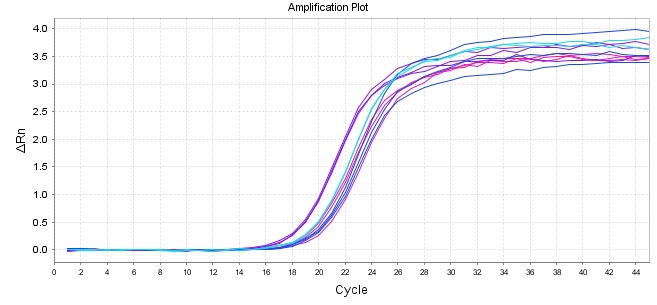
**

1. CRT-Amplification Plot

**2) Melt Curve**

**
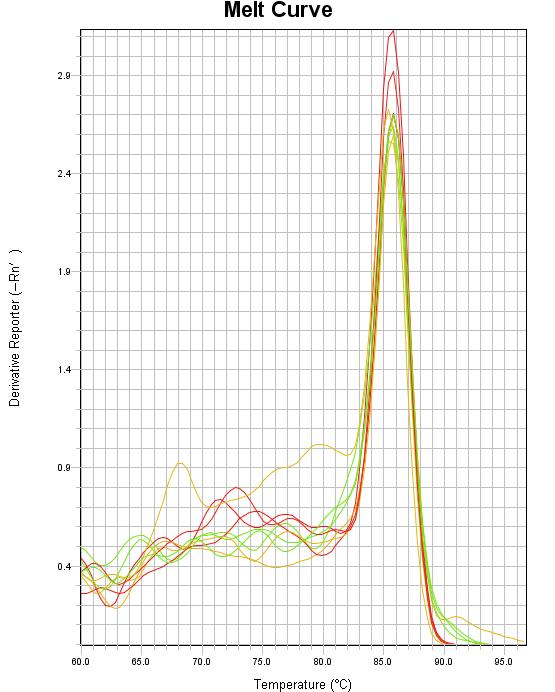
**

1. mus-GAPDH-Melt Curve

##
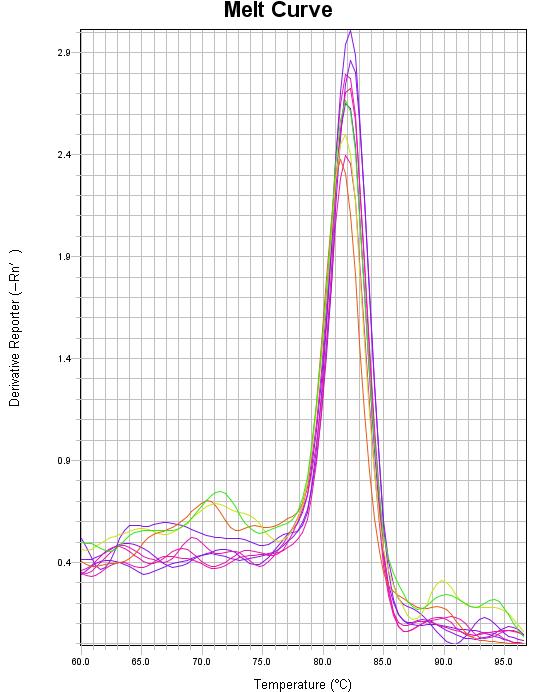


(2)cat-Melt Curve

##
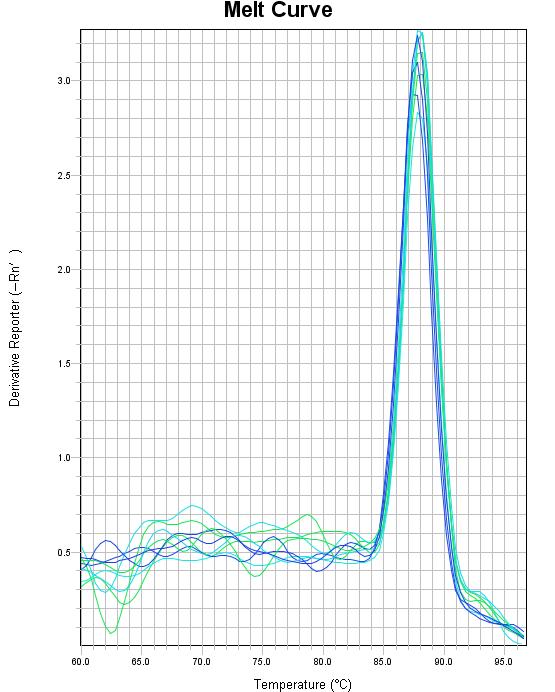


(3)GSTP1-Melt Curve

**
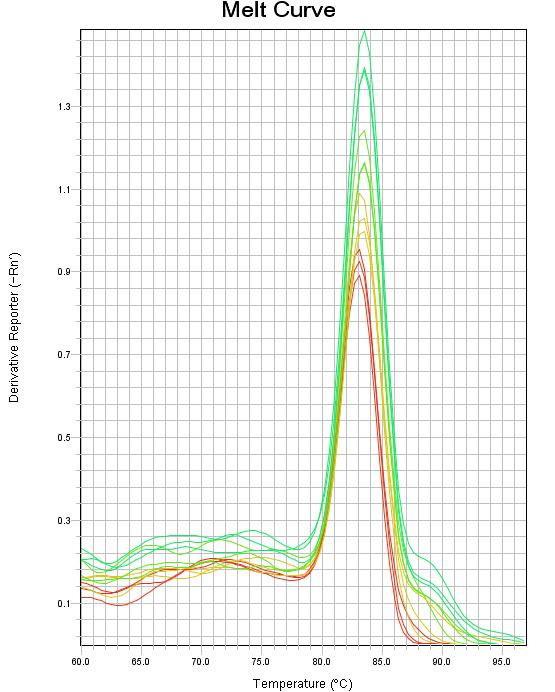
**

(4)CRT-Melt Curve

**3) Data analysis**

CAT：

| Sample | Target gene Ct | GAPDH Ct | △Ct | △△Ct | RQ(2^-△△Ct^) | Average | Stdevp |
| --- | --- | --- | --- | --- | --- | --- | --- |
| Control | 19.04 | 16.05 | 2.98 | 0 | 1 | 1 |  |
|  | 18.95 | 15.81 | 3.13 | 0 | 1 |  |  |
|  | 18.87 | 15.85 | 3.02 | 0 | 1 |  |  |
| <50 μGy/h | 18.81 | 15.30 | 3.52 | 0.53 | 1.25 | 1.07 | 0.058 |
|  | 18.61 | 15.25 | 3.36 | 0.23 | 1.03 |  |  |
|  | 18.60 | 15.46 | 3.14 | 0.12 | 0.92 |  |  |
| 50–500 μGy/h | 21.01 | 15.95 | 5.06 | 2.07 | 1.12 | 1.13 | 0.016 |
|  | 20.95 | 15.99 | 4.95 | 1.82 | 1.14 |  |  |
|  | 20.97 | 16.22 | 4.75 | 1.73 | 1.15 |  |  |
| 500–1000 μGy/h | 18.84 | 16.63 | 2.21 | -0.78 | 1.72 | 2.07 | 0.326 |
|  | 18.84 | 16.82 | 2.01 | -1.12 | 2.17 |  |  |
|  | 18.76 | 16.97 | 1.79 | -1.23 | 2.35 |  |  |

GSTP1：

| Sample | Target gene Ct | Actin Ct | △Ct | △△Ct | RQ(2^-△△Ct^) | Average | Stdevp |
| --- | --- | --- | --- | --- | --- | --- | --- |
| Control | 25.75 | 16.05 | 9.69 | 0 | 1 | 1 |  |
|  | 25.92 | 15.81 | 10.10 | 0 | 1 |  |  |
|  | 25.82 | 15.85 | 9.96 | 0 | 1 |  |  |
| <50 μGy/h | 23.91 | 15.30 | 10.61 | -1.17 | 2.35 | 1.68 | 0.14 |
|  | 24.92 | 15.25 | 9.67 | -0.43 | 1.35 |  |  |
|  | 25.11 | 15.46 | 9.64 | -0.32 | 1.24 |  |  |
| 50–500 μGy/h | 24.85 | 15.95 | 8.90 | -0.79 | 1.73 | 2.03 | 0.25 |
|  | 24.9 | 15.99 | 8.96 | -1.13 | 2.19 |  |  |
|  | 25.08 | 16.22 | 8.86 | -1.10 | 2.15 |  |  |
| 500–1000 μGy/h | 25.68 | 16.63 | 8.99 | -0.70 | 1.63 | 2.14 | 0.44 |
|  | 25.66 | 16.82 | 8.83 | -1.27 | 2.41 |  |  |
|  | 25.67 | 16.97 | 8.70 | -1.26 | 2.40 |  |  |

CRT：

| Sample | Target gene Ct | Actin Ct | △Ct | △△Ct | RQ(2^-△△Ct^) | Average | Stdevp |
| --- | --- | --- | --- | --- | --- | --- | --- |
| Control | 25.64 | 16.05 | 9.59 | 0 | 1 | 1 |  |
|  | 25.53 | 15.81 | 9.71 | 0 | 1 |  |  |
|  | 25.43 | 15.85 | 9.57 | 0 | 1 |  |  |
| <50 μGy/h | 25.45 | 16.30 | 9.15 | -0.48 | 1.38 | 1.76 | 0.125 |
|  | 25.35 | 16.25 | 9.10 | -0.55 | 1.47 |  |  |
|  | 25.14 | 16.46 | 8.68 | -1.09 | 2.10 |  |  |
| 50–500 μGy/h | 24.98 | 15.95 | 9.03 | -0.55 | 1.47 | 1.86 | 0.348 |
|  | 24.73 | 15.99 | 8.73 | -0.97 | 1.96 |  |  |
|  | 24.69 | 16.22 | 8.47 | -1.10 | 2.14 |  |  |
| 500–1000 μGy/h | 25.74 | 16.63 | 9.10 | -0.48 | 1.39 | 1.81 | 0.385 |
|  | 25.60 | 16.82 | 8.78 | -0.93 | 1.90 |  |  |
|  | 25.44 | 16.97 | 8.46 | -1.10 | 2.15 |  |  |
