# Supplementary material for: Up-regulation of calreticulin in mouse liver tissues after long-term irradiation with low-dose-rate gamma rays
Source: PLoS One. 2017 Sep 20;12(9):e0182671. doi: 10.1371/journal.pone.0182671 (PMC5607120; doi:10.1371/journal.pone.0182671)
Supplement: S4 File — (DOCX) [file pone.0182671.s004.docx]

Gray value of CRT, GSTP1, CAT

1) CRT

| Sample | CRT | GAPDH | CRT/GAPDH | mean | Average | Sd |
| --- | --- | --- | --- | --- | --- | --- |
| Control | 2676.44 | 4580.86 | 0.58 | 0.59 | 100 |  |
|  | 3254.89 | 5523.21 | 0.55 |  |  |  |
|  | 2489.56 | 4043.11 | 0.62 |  |  |  |
| <50 μGy/h | 3454.89 | 3056.89 | 1.01 | 1.07 | 181.35 | 4.23 |
|  | 4432.76 | 4005.36 | 1.10 |  |  |  |
|  | 3215.99 | 2944.55 | 1.09 |  |  |  |
| 50–500 μGy/h | 3512.78 | 3087.23 | 1.11 | 1.12 | 189.83 | 3.48 |
|  | 4532.19 | 4123.32 | 1.10 |  |  |  |
|  | 3321.89 | 2876.90 | 1.15 |  |  |  |
| 500–1000 μGy/h | 3457.65 | 5056.21 | 0.68 | 0.66 | 111.86 | 8.23 |
|  | 4682.22 | 7543.77 | 0.62 |  |  |  |
|  | 3187.34 | 4586.12 | 0.69 |  |  |  |

2) GSTP1

| Sample | GSTP1 | GAPDH | GSTP1/GAPDH | mean | Average | Sd |
| --- | --- | --- | --- | --- | --- | --- |
| Control | 876.44 | 4280.86 | 0.20 | 0.23 | 100 |  |
|  | 1043.89 | 5723.21 | 0.18 |  |  |  |
|  | 1489.56 | 4943.11 | 0.30 |  |  |  |
| <50 μGy/h | 2454.89 | 4856.89 | 0.51 | 0.51 | 221.73 | 8.76 |
|  | 3632.76 | 7001.63 | 0.52 |  |  |  |
|  | 3815.99 | 7544.73 | 0.51 |  |  |  |

3) CAT

| Sample | CAT | GAPDH | CAT/GAPDH | mean | Average | Sd |
| --- | --- | --- | --- | --- | --- | --- |
| Control | 658.19 | 3245.87 | 0.20 | 0.20 | 100 |  |
|  | 965.12 | 4672.13 | 0.21 |  |  |  |
|  | 1244.65 | 6219.07 | 0.20 |  |  |  |
| 500–1000 μGy/h | 3757.45 | 6076.99 | 0.62 | 0.61 | 305.10 | 12.89 |
|  | 4383.28 | 7043.87 | 0.62 |  |  |  |
|  | 3219.51 | 5417.11 | 0.59 |  |  |  |
